# Integrative multi-omics reveals microbial genomic variants driving altered host-microbe interactions in autism spectrum disorder

**DOI:** 10.1016/j.xcrm.2025.102516

**Published:** 2025-12-19

**Authors:** Wanning Chen, Xinjun Wang, Ruixin Zhu, Wenxing Gao, Liwen Tao, Rong Yang, Qing Wei, Yiming Zhang, Yujiao Gong, Hui Zhong, Linsheng Huang, Xinyue Zhu, Yuwei Yang, Linjuan Zhang, Lin Wan, Guang Yang, Yan Li, Na Jiao, Jifeng Wang, Huanlong Qin, Lixin Zhu

**Affiliations:** 1 Shanghai Key Laboratory of Maternal Fetal Medicine, Shanghai Institute of Maternal-Fetal Medicine and Gynecologic Oncology, Clinical and Translation Research Center, Shanghai First Maternity and Infant Hospital, School of Life Sciences and Technology, Tongji University, Shanghai 200092, P.R. China; 2 Institutes of Biomedical Sciences, School of Life Sciences, Inner Mongolia University, Hohhot 010070, P.R. China; 3 Suzhou Key Laboratory of Gut Microecology, Affiliated Suzhou Hospital of Nanjing Medical University, Suzhou Municipal Hospital, Gusu School, Nanjing Medical University, Suzhou 215000, P.R. China; 4 School of Medicine, Tongji University, Shanghai 200092, P.R. China; 5 State Key Laboratory of Genetic Engineering, Fudan Microbiome Center, School of Life Sciences, Fudan University, Shanghai 200438, P.R. China; 6 Department of Pediatrics, Shanghai Tenth People’s Hospital, Tongji University School of Medicine, Shanghai 200072, P.R. China; 7 Department of Pathology, Shanghai Tenth People’s Hospital, Shanghai 200072, P.R. China; 8 Department of Stomatology, Shanghai Tenth People’s Hospital, School of Medicine, Tongji University, Shanghai 200092, P.R. China; 9 Center for Brain Science, The First Affiliated Hospital of Xi’an Jiaotong University, Xi’an 710061, P.R. China; 10 Senior Department of Pediatrics, Chinese PLA General Hospital, Beijing 100700, P.R. China; 11 Division of Epidemiology, Department of Medicine, Vanderbilt Epidemiology Center, Vanderbilt-Ingram Cancer Center, Vanderbilt University Medical Center, Nashville, TN, USA

**Keywords:** autism spectrum disorder, microbiome, metabolomics, genomic variants, mediation analysis

## Abstract

Emerging evidence links the gut microbiome to autism spectrum disorder (ASD), yet the role of microbial genomic variation remains underexplored. We generated a large-scale metagenomic and metabolomic dataset from over 1,100 children, integrating public datasets, to characterize ASD-associated microbial changes. We identified 35 species, 213 genes, 28 pathways, and 99 metabolites, alongside 1,369 single-nucleotide variants, 233 insertions/deletions, and 195 structural variants with differential abundance. Profiling of microbial genomic variation revealed 33 species and 196 enzymes lacking abundance differences, yet exhibiting significant sequence variation. Integrated analysis of microbial variants and metabolites uncovered 357 neurological associations, with mediation analysis showing that several metabolites link microbial variants to the ASD phenotype. Importantly, diagnostic models incorporating microbial variant and/or metabolite features achieved superior performance and generalizability. Our findings highlight microbial genomic variation as a critical, previously overlooked dimension of ASD-associated dysbiosis, offering valuable insights for diagnosis and mechanistic studies.

## Highlights

• Large-scale metagenomic and metabolomic integration across >1,100 children with ASD
• Microbial genomic variants define a distinct layer of ASD-associated dysbiosis
• Variant-driven metabolic alterations link microbial genetics to ASD pathophysiology
• A 20-marker multi-omics panel enables accurate, non-invasive ASD diagnosis

## Graphical abstract

**Figure:**
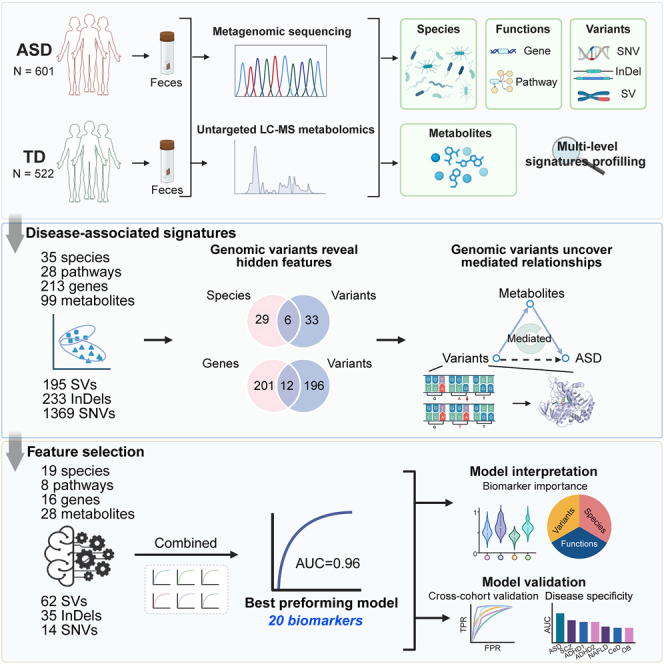

## Teaser

Integrating gut metagenomic and metabolomic profiles from over 1,100 children, Chen et al. identify microbial genomic variants and metabolic shifts associated with autism spectrum disorder, providing a multi-layered signature for precise, non-invasive diagnosis and mechanistic understanding.

## Introduction

Autism spectrum disorder (ASD) is a complex neurodevelopmental condition characterized by deficits in social communication and the presence of restricted, repetitive behaviors. ASD presents a growing public health challenge that urgently requires better understanding of its underlying etiology and effective diagnostic strategies.^1^^,^^2^ While twin studies^3^ and large-scale sequencing^4^ efforts have identified numerous host genetic risk factors associated with ASD, these variants explain only a limited proportion of cases,^5^^,^^6^ highlighting a substantial contribution from environmental contributors.

Among these, the gut microbiome has recently drawn considerable attention due to its potential role in modulating neurodevelopment through the gut-brain axis.^7^^,^^8^^,^^9^^,^^10^ This interest is further supported by clinical observations that a significant proportion of children with ASD also experience gastrointestinal comorbidities.^11^ Building on this connection, accumulating evidence from animal studies^12^^,^^13^ and human metagenomic analyses^14^^,^^15^^,^^16^ have consistently demonstrated associations between alterations in gut microbiota composition and ASD-related behavioral phenotypes. Supporting a causal role, preclinical research has shown that fecal microbiota transplantation (FMT) from individuals with ASD can induce autistic-like behaviors in recipient mice,^12^ while FMT from healthy donors to children with ASD has resulted in symptomatic improvements.^17^^,^^18^

However, current microbiome research in ASD has predominantly focused on taxonomic composition and microbial functional capacities,^14^^,^^15^^,^^19^^,^^20^^,^^21^ largely overlooking extensive microbial genomic variation.^22^ Such genomic variations, including structural variants (SVs, ≥50 bp),^23^^,^^24^^,^^25^ insertions and deletions (InDels, <50 bp),^26^ and single-nucleotide variants (SNVs),^24^^,^^27^ represent an essential yet underexplored layer of microbial genetic diversity. These genetic alterations not only exhibit potential as discriminative biomarkers across samples^24^^,^^26^^,^^28^^,^^29^ but also can substantially impact microbial functions, including metabolic capabilities and interactions with the host.^23^^,^^27^

In line with this notion, metabolomic profiling analyses have already revealed distinct metabolic signatures in ASD,^30^^,^^31^^,^^32^^,^^33^^,^^34^ which are frequently observed alongside specific alterations in the gut microbiome. Nevertheless, systematically integrating microbial genomic variation with metabolic pathways remains understudied. Clarifying the variant-metabolite-ASD connection could substantially advance our understanding of ASD pathophysiology and ultimately guide the development of microbiome-based diagnostic and therapeutic approaches.

In this study, we performed integrative metagenomic and untargeted metabolomic profiling of in-house stool samples from 270 children, including those with ASD and typically developing (TD) controls. In addition to taxonomic and functional alterations, we systematically characterized microbial genomic variants and their putative associations with host through metabolites. Finally, we constructed a multi-layered diagnostic model incorporating microbial and metabolic features, achieving high predictive accuracy and disease specificity, and validated its performance using an independent set of 987 publicly available fecal metagenomes.

## Results

### Cohort design and multi-omics data generation

**Figure 1:**
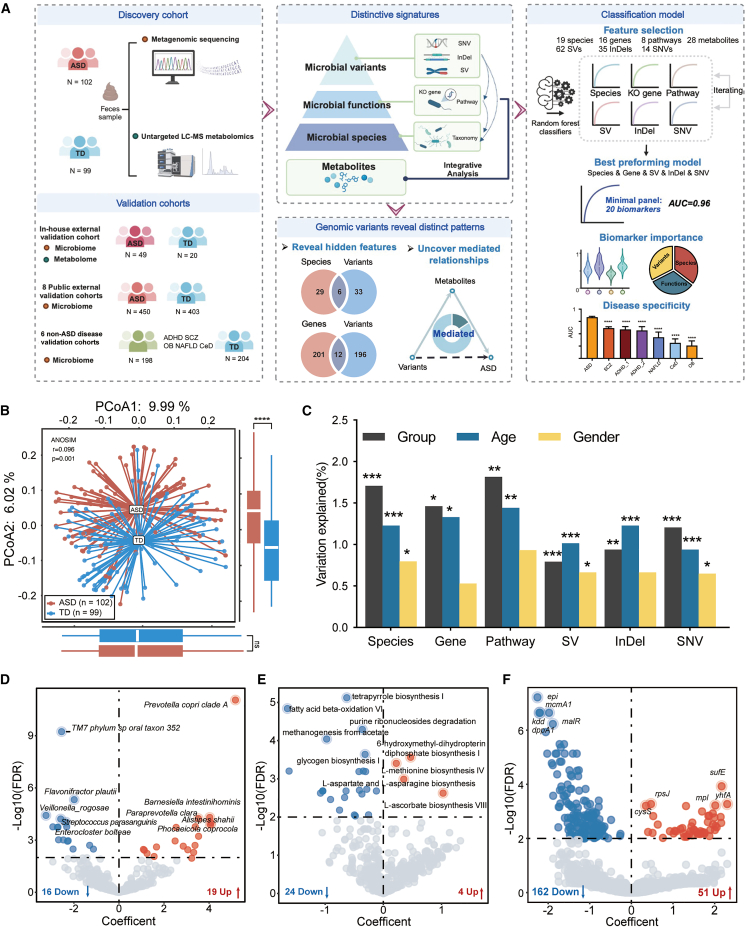
Overview of cohort design and gut microbiome alterations in ASD (A) Schematic summary of study design and sample composition across discovery, validation, and external cohorts (*N* = sample size). (B) PCoA based on averaged beta diversity across species, gene, and pathway, showing compositional differences between ASD and TD groups. Overall separation was assessed by ANOSIM. Differences in PCoA1 and PCoA2 scores were tested using the Wilcoxon test. ∗∗∗∗*p* < 0.0001; ns, not significant. (C) Proportion of variance in microbial features explained by group, age, and gender, assessed by multivariate PERMANOVA. ∗*p* < 0.05; ∗∗*p* < 0.01; ∗∗∗*p* < 0.001. (D–F) Volcano plots showing differential associations between microbial species (D), functional pathways (E), and gene families (F) and ASD status, analyzed using MaAsLin2 with adjustment for significant covariates. Features with an FDR < 0.01 were considered significant and are highlighted in red (ASD enriched) or blue (ASD depleted). Top-ranked features are labeled by name. See also Figure S1, Data S1 and S2.

A total of 270 children were recruited in this study. All participants underwent detailed clinical assessments and provided fecal samples for integrative multi-omics profiling. The discovery cohort consisted of 102 children diagnosed with ASD and 99 age- and gender-matched TD controls, from whom stool samples were subjected to shotgun metagenomic sequencing and untargeted liquid chromatography-mass spectrometry-based metabolomic analysis. An independent in-house validation cohort (ASD: 49; TD: 20) with paired microbiome-metabolome data was recruited from the same clinical center to assess model robustness and generalizability, following the identical analytical protocols. To further validate our findings across broader populations, we included 853 publicly available fecal metagenomic datasets from children across multiple geographic regions, representing one of the largest ASD-focused gut microbiome validation cohorts to date. Additionally, to assess panel’s specificity, we also incorporated six non-ASD cohorts with known microbiota-related conditions: attention-deficit/hyperactivity disorder (ADHD1, *n* = 78; ADHD2, *n* = 50), schizophrenia (SCZ, *n* = 171), celiac disease (CeD, *n* = 39), non-alcoholic fatty liver disease (NAFLD, *n* = 24), and obesity (OB, *n* = 40) (Data S1, Figure 1A).

All metagenomic data across cohorts were processed under a unified computational framework and annotated across six analytical layers, including microbial taxonomic composition, gene abundance, pathway profiles, and three classes of genomic variation (SVs, InDels, and SNVs). These multi-dimensional microbial features were subsequently integrated with metabolomic data to identify ASD-associated molecular signatures.

### Taxonomic dysbiosis and functional shifts in the gut microbiota of individuals with ASD

In the discovery cohort, we identified 2,838 microbial taxa, spanning two kingdoms, 14 phyla, 177 classes, 203 orders, 248 families, 744 genera, and 1,450 species. Compared to TD children, individuals with ASD exhibited a significantly lower species-level Shannon diversity index (Figure S1A). In addition, the overall microbial community composition differed significantly between groups (analysis of similarities [ANOSIM], *p* = 0.001, r = 0.089; Figure S1B). Functional profiling revealed 5,543 microbial genes and 510 metabolic pathways, with modest but significant group-level differences in both gene (ANOSIM, *p* = 0.001, r = 0.040) and pathway (ANOSIM, *p* = 0.001, r = 0.047) profiles (Figures S1B and S1C). Notably, integrating taxonomic and functional distance matrices enhanced group discrimination (ANOSIM, r = 0.096; Figure 1B), highlighting the added value of combining multi-layer microbiome features.

Group status accounted for 1.71% of the inter-individual variance in microbiome composition—exceeding the variance explained by age or gender (Figure 1C). After adjusting for potential confounders, we identified 35 species with differential abundance in ASD, including 19 enriched and 16 depleted species (false discovery rate [FDR] <0.01; Figure 1D; Data S2). ASD-enriched species included *Prevotella copri*, *Klebsiella pneumoniae*, and *Alistipes putredinis*, all previously implicated in neuroinflammation and compromised intestinal barrier function.^35^^,^^36^ Several understudied species, such as *Phocaeicola coprocola* and *Oscillibacter* sp. *ER4*, were also elevated. Conversely, ASD-depleted species included short-chain fatty acid (SCFA) producers within the Clostridia class (e.g., *Clostridium butyricum* and *Faecalicatena contorta*) and oral-derived species (e.g., *Streptococcus australis* and *Veillonella parvula*), suggesting alterations in both microbial metabolic capacity and ecological origin.

At the functional level, 213 microbial genes and 28 metabolic pathways were significantly altered in ASD (FDR <0.01; Figures 1E and 1F; Data S2). Notably, pathways involved in amino acid biosynthesis—such as L-aspartate, L-asparagine, and L-methionine biosynthesis—were enriched in ASD. In contrast, multiple SCFA-generating fermentation pathways (e.g., succinate to butanoate, pyruvate to butanoate, and Stickland fermentation) were markedly depleted, consistent with the reduced abundance of butyrate-producing taxa. Additional impairments were observed in peptidoglycan biosynthesis, methanogenesis, and various vitamin and carbohydrate metabolism pathways, reflecting broader disruptions in microbial functional potential and host-microbe metabolic interactions.

### Profiling of bacterial genomic variation in ASD gut microbiota

**Figure 2:**
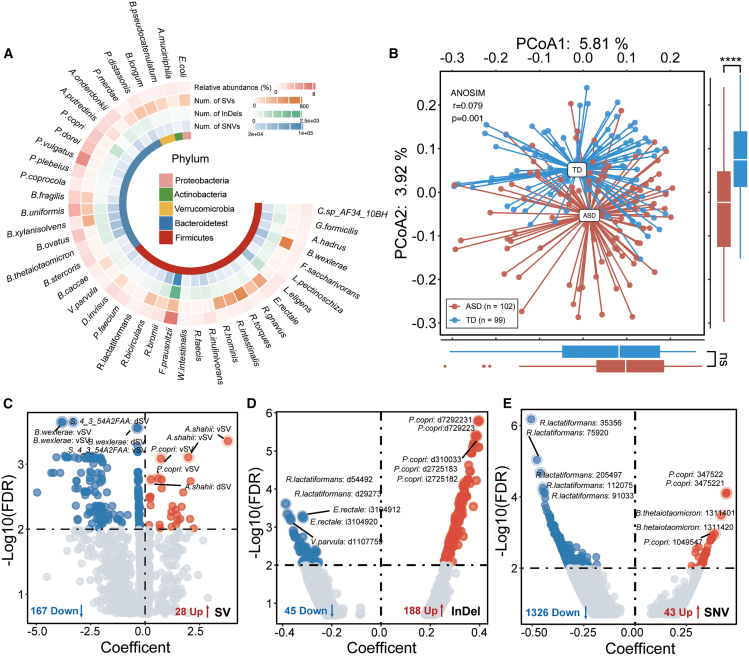
Overview of microbial genomic variants associated with ASD (A) Number of SVs, InDels, and SNVs of each species, alongside the corresponding relative abundance of each corresponding species. (B) PCoA based on averaged beta diversity across SV, InDel, and SNV, revealing global differences in microbial variant profiles between ASD and TD groups. Overall separation was assessed by ANOSIM. Differences in PCoA1 and PCoA2 scores were tested using the Wilcoxon test. ∗∗∗∗*p* < 0.0001; ns, not significant, (C–E) Volcano plots showing differential associations between microbial SVs (C), InDels (D), and SNVs (E) and ASD status, analyzed using MaAsLin2 with adjustment for significant covariates. Features with an FDR < 0.01 were considered significant and are highlighted in red (ASD enriched) or blue (ASD depleted). Top-ranked features are labeled by name. See also Data S2.

To assess the potential involvement of microbial genetic variation in ASD, we systematically characterized the prevalence and distribution of SVs, InDels, and SNVs in gut microbial species with sufficient metagenomic coverage, comparing ASD and TD groups. (Figure 2A; Data S3 and S4). While both age and gender contributed to inter-individual variation in microbial variant profiles, ASD diagnosis consistently explained a greater proportion of variance across all three variant types (Figure 1B). When dissimilarity matrices from SVs, InDels, and SNVs were integrated, the combined variant-level profile yielded improved discrimination between ASD and TD groups (ANOSIM, *p* = 0.001, r = 0.079), underscoring the additive contribution of multi-scale genomic variation to disease-associated microbial signatures.

At the SV level, we identified 6,137 SVs across 63 species, including 1,890 variable SVs (vSVs) and 4,247 deletion SVs (dSVs), ranging from 1 to 330 SVs per species (Figure S2A; Data S3). Among these, 195 SVs, comprising 67 dSVs and 128 vSVs, were significantly different in abundance between ASD and TD children (FDR <0.01, Figure 2C; Data S2 and S3). The average length of these differential SVs was 4.83 kb, reflecting their potential to encompass multiple genes or regulatory elements and thereby influence microbial function. In total, these ASD-associated SVs mapped to 318 distinct genomic regions. Interestingly, although dSVs were globally more prevalent, vSVs made up a larger proportion of the SVs that were significantly associated with ASD. This suggests that changes in genomic variability, rather than just gene loss, may be a more prominent feature in ASD. Moreover, differential SVs were particularly enriched in *Subdoligranulum* sp. *4_3_54A2FAAI* (91, 46.7%) and *Blautia wexlerae* (60, 30.8%), both members of the Firmicutes phylum (Figure S3A), which have been previously linked to SCFA metabolism and mucosal health.

At the InDel level, we detected a total of 116,016 InDels across 42 high-coverage reference species (Data S4), consisting of 57,178 deletions and 58,838 insertions, with relatively balanced distribution across variant types (Figure S2B; Data S2). Furthermore, we identified 233 ASD-associated InDels (FDR <0.01), comprising 120 deletions and 113 insertions from 13 species, with average length of 2.82 bp (Figure 2D). Strikingly, 86.3% of these differential InDels (*n* = 201) were localized within *P*. *copri* (Figure S3B).

For SNV, we annotated 751,468 positions across the same 42 species, applying a prevalence filter (>40%) to ensure robust comparisons (Figure S2C; Data S4). A total of 1,369 SNVs from 26 species were identified as differentially abundant between ASD and TD (Figure 2E; Data S2). Notably, the vast majority of differential SNVs were concentrated in *Ruthenibacterium lactatiformans* (999, 73.0%) and *V. parvula* (228, 16.7%) (Figure S3C). Our findings demonstrate widespread genomic variation in ASD-associated gut microbiota, providing a critical layer of functional diversity that may shape the biochemical environment of the ASD gut.

### Microbial genomic variants identify ASD-associated patterns uncaptured by taxonomic or functional profiles

**Figure 3:**
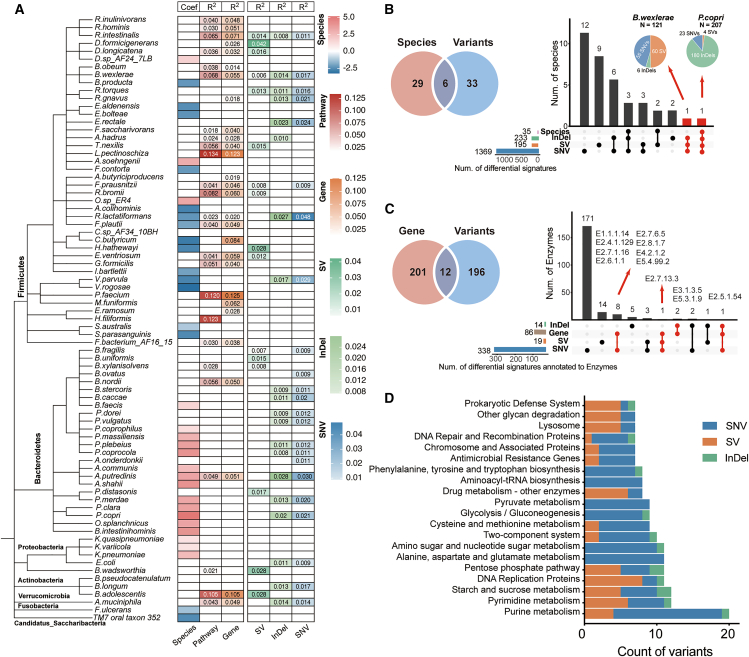
Genomic variants reveal ASD-associated patterns beyond taxonomic and functional differences (A) Heatmap summarizing ASD-related signals at the microbial species level across multiple data layers. The first column indicates species-level differential abundance (MaAslin2), with effect sizes (Coef, coefficient values) shown for significant features. The remaining five columns represent the proportion of variance in compositional dissimilarity explained by ASD diagnosis (R^2^ from PERMANOVA, 999 permutations) at the pathway, gene, SV, InDel, and SNV levels, respectively. Statistical significance is marked with R^2^ (FDR <0.05). (B) Left: Venn diagram illustrating the overlap between species identified as differentially abundant and those harboring ASD-associated genomic variants. Right: an UpSet plot quantifying the intersection of species across different types of differential variants (SVs, InDels, SNVs). Species shared across all three variant types are highlighted, with pie charts indicating the distribution of differential variants within each species. (C) Left: Venn diagram illustrating the overlap between genes identified as differentially abundant and those harboring ASD-associated genomic variants. Right: an UpSet plot quantifying the intersection of genes across different types of differential variants (SVs, InDels, and SNVs). Differential genes and those containing ASD-associated variants are highlighted. (D) Distribution of differential genomic variants across microbial pathways. Top 20 pathways harboring the most variants are shown. See also Datas S5 and S6.

To determine whether microbial genomic variation reveals additional ASD-associated patterns beyond differential species and functions, we first constructed population structures for each species based on their genomic variation profiles. Notably, several species without differential abundance in ASD—such as *R*. *intestinalis* and *B*. *wexlerae*—exhibited significant associations between the genomic or functional alterations and ASD status (permutational multivariate analysis of variance [PERMANOVA], FDR <0.05). In addition, although functional shifts were often accompanied by underlying genomic changes (16 species), a distinct set of species demonstrated ASD-associated variation exclusively at the genomic variation level (11 species) (Figure 3A).

In closer examinations of ASD-specific features, 33 species exhibited no significant differences in relative abundance between ASD and TD groups but carried a substantial number of ASD-associated genomic variants (Figure 3B). Among them, 12 species exhibited ASD-associated variation exclusively at the SNV level (e.g., *Bacteroides thetaiotaomicron* and *Faecalibacterium prausnitzii*), 9 species at the SV level (e.g., *Subdoligranulum* sp. *4_3_54A2FAA* and *Alistipes shahii*), and 2 species at InDel level (*Alistipes onderdonkii* and *Fusicatenibacter saccharivorans*). Several taxa carried multiple types of differential variants; e.g., *B*. *wexlerae* harbored 121 ASD-associated variants, primarily SNVs and SVs. Notably, the extensive genomic alterations observed in *P. copri*, encompassing all three variant types alongside altered abundance, underscores its potential functional relevance in ASD pathophysiology.

To further explore potential functional implications, we mapped the variant loci to genomic regions and integrated them with enzyme annotations at the gene level (Data S5). Although most variants were located outside protein-coding regions, we identified a total of 371 ASD-associated variants that were located in the open reading frame of 208 enzymes, notably including 196 that were overlooked by traditional gene abundance comparisons. Among these, 171, 14, and 5 enzymes were exclusively associated with SNVs, SVs, and InDels, respectively. To highlight enzymes with potential biological significance in ASD, we prioritized those that not only carried genomic variants but also showed significant differences in gene abundance between ASD and TD children. This yielded 12 enzymes of particular interest. Notably, 3-deoxy-7-phosphoheptulonate synthase (EC 2.5.1.54) and histidine kinase (EC 2.7.13.3) were impacted by multiple variant types, suggesting strong genomic pressure on their regulation. In addition, 10 enzymes were influenced by a single variant type, yet they also showed altered abundance. Two of these—EC 3.1.3.5 (alkaline phosphatase) and EC 5.3.1.9 (glucose-6-phosphate isomerase)—were affected by InDels and are involved in carbohydrate metabolism. Eight others were impacted by SNVs and mapped to a broad array of functional categories, including nucleotide metabolism (EC 1.1.1.14), cell wall biosynthesis (EC 2.4.1.129), amino acid metabolism (EC 2.6.1.1), pentose metabolism (EC 2.7.1.16), purine metabolism (EC 2.7.6.5), iron-sulfur cluster assembly (EC 2.8.1.7), tricarboxylic acid cycle (EC 4.2.1.2), and propionate metabolism (EC 5.4.99.2). These enzymes are central to energy production, biosynthetic metabolism, and bacterial signal transduction, indicating that microbial variants may directly influence core physiological functions in the ASD gut microbiome (Figure 3C).

Consistently, pathway-level annotation further highlighted the concentration of variants in core microbial metabolic processes, including purine and pyrimidine metabolism, central carbon metabolism (e.g., glycolysis, pentose phosphate, and starch/sucrose metabolism), and amino acid biosynthesis (Figure 3D). In parallel, we observed enrichment of differential variants in pathways involved in genetic information processing (e.g., DNA replication and repair) and environmental sensing (e.g., two-component systems) (Figure 3D). Notably, 13 microbial pathways—including purine metabolism, pyrimidine metabolism, and the two-component system—were impacted by all three variant types, underscoring their vulnerability to broad-spectrum mutational pressures (Data S6).

Together, these results demonstrate that genomic variant profiling captures a distinct layer of ASD-associated microbial alterations, many of which are invisible to conventional taxonomic or gene-based analyses.

### Microbial abundance and genomic changes are associated with fecal metabolites

**Figure 4:**
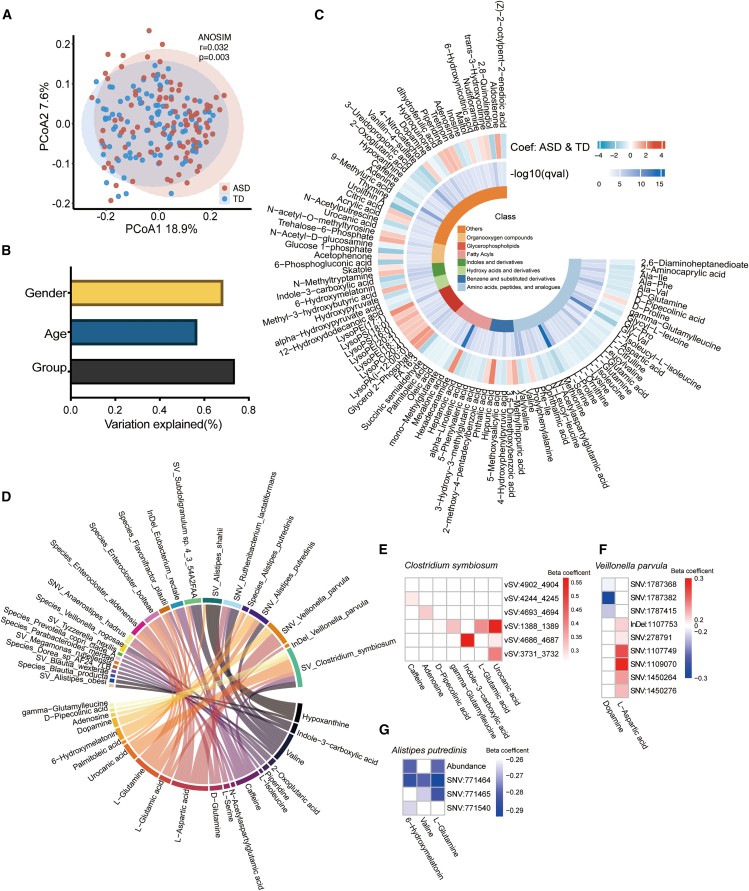
Differential fecal metabolites in ASD and associations between neuro-related metabolites and microbial signatures (A) PCoA shows significant differences in overall fecal metabolite composition between groups (*p* = 0.003). (B) Proportion of variance in individual metabolite features explained by clinical phenotypes, based on multivariate PERMANOVA. (C) Differentially abundant fecal metabolites between ASD and TD groups. Red indicates metabolites enriched in ASD; blue indicates depletion in ASD. Coef, regression coefficient. (D) Significant associations between neuro-related metabolites and microbial signatures (FDR <0.05). (E–G) Heatmap of associations between neuro-related metabolites and microbial signatures in *C. symbiosum* (E), *V. parvula* (F), and *A. putredinis* (G). See also Datas S7 and S8.

We next explored fecal metabolic alterations in ASD using untargeted metabolomics (Figure 1A). Principal coordinate analyses (PCoA) analysis revealed a modest yet significant separation in overall metabolic profiles between ASD and TD children (Figure 4A), with ASD status accounting for a substantial proportion of the variance in specific metabolites (Figure 4B). Differential abundance analysis identified 99 significantly altered fecal metabolites (68 down-regulated and 31 up-regulated in ASD) (Figure 4C; Data S7). Strikingly, many of these metabolites are known to be involved in neuroactive signaling or inflammation. Key excitatory neurotransmitters such as L-glutamic acid and L-aspartic acid, as well as GABAergic pathway-related metabolites (e.g., N-acetylaspartylglutamic acid and succinic semialdehyde), were significantly reduced in ASD. Monoamine-related compounds, including dopamine, caffeine, and 6-hydroxymelatonin, also showed marked depletion. Additional neuroactive metabolites such as N-methyltryptamine and L-serine were similarly downregulated, indicating widespread perturbation of neurotransmitter metabolism. In contrast, ASD children exhibited elevated levels of microbial-host co-metabolites and xenobiotic degradation products, including hippuric acid and phthalic acid.

To gain insight into the metabolic interface between the gut microbiota and host physiology, we systematically integrated microbial features and fecal metabolites. By performing a regression-based correlation analysis between significantly altered microbiome variables and fecal metabolites, we detected a total of 1,154 significant associations between 529 microbial features and 77 metabolites, including 59 associations with microbial abundance, 116 associations with microbial SVs, 16 associations with microbial InDels, 251 associations with SNVs, and 712 associations with microbial functions (FDR <0.05) (Figure S4; Data S8).

At the species abundance level, *Enterocloster aldenensis* showed the highest number of associations, followed by *Enterocloster bolteae*, *A. putredinis*. *Flavonifractor plautii,* and *P. copri*. When microbial variants were also considered, *R. lactatiformans* emerged as the most highly associated species, followed by *Subdoligranulum* sp. *4_3_54A2FAA*, *V. parvula*, *A. putredinis*, and *B. wexlerae* (Figure S4). Notably, 17.9% (207 of 1,154) of the microbial associations with metabolites were related to SNVs of *R*. *lactatiformans*. Among these, there were 122 negative associations with lysophospholipids, including LysoPA(i-12:0/0:0). In particular, SNVs from *R*. *lactatiformans* that negatively correlated with LysoPA(i-12:0/0:0) were enriched in genomic regions encoding enzymes such as L-serine dehydratase, inositol-1-monophosphatase, and acetaldehyde dehydrogenase, which are involved in precursor pathways for glycerophospholipid biosynthesis and inositol signaling turnover (Data S8).

Interestingly, many metabolites associated with the microbiome are already known to be related to the gut microbiome. For example, several hydroxy acids and derivatives (Figure S4) are known to be by-products or intermediates in microbial fermentation. Hydroxypyruvic acid—a β-hydroxy acid involved in glyoxylate and serine metabolism—showed strong positive association with a vSV (2079_2081 kb) from *B*. *wexlerae* (β = 0.32, FDR = 0.0010). Similarly, alpha-hydroxyisobutyric acid, a valine catabolite, was positively associated with SNV-level or SV-level features from *Clostridium symbiosum*, *V. parvula*, and *TM7 phylum sp. Oral taxon 352* (Data S8).

Among the most prominent metabolite categories, neuro-related metabolite accounted for a substantial portion of significant associations: 357 associations for 23 neuro-related metabolites (Figure 4D; Data S8). *C. symbiosum* showed the highest number of associations (Figure 4E), followed by *V. parvula* (Figure 4F) and *A. putredinis* (Figure 4G). Several vSVs of *C. symbiosum* were positively linked to excitatory neurotransmitters, including L-glutamic acid, adenosine, caffeine, and indole-3-carboxylic acid. Notably, the strongest association was observed between vSV:4686_4687 kb and indole-3-carboxylic acid (β = 0.57, FDR = 2.1 × 10^−15^), a microbial tryptophan-derived metabolite implicated in gut-brain signaling.^37^^,^^38^ Additionally, this strain was also linked to elevated levels of urocanic acid, a histidine derivative with neuro-immune relevance,^39^ through multiple independent vSVs (e.g., vSV:3731_3732 kb, β = 0.40, FDR = 2.8 × 10^−6^; vSV:1388_1389 kb, β = 0.56, FDR = 6.6 × 10^−15^). *V. parvula* exhibited multiple SNV-level associations, particularly with dopamine and L-aspartic acid, and *A. putredinis* showed consistent negative associations with 6-hydroxymelatonin and L-glutamine. These findings confirmed that variant-defined functional shifts contribute substantially to altered metabolic circuits in ASD (Figure S5).

### The microbiome contributes to host phenotypic changes through metabolites

**Figure 5:**
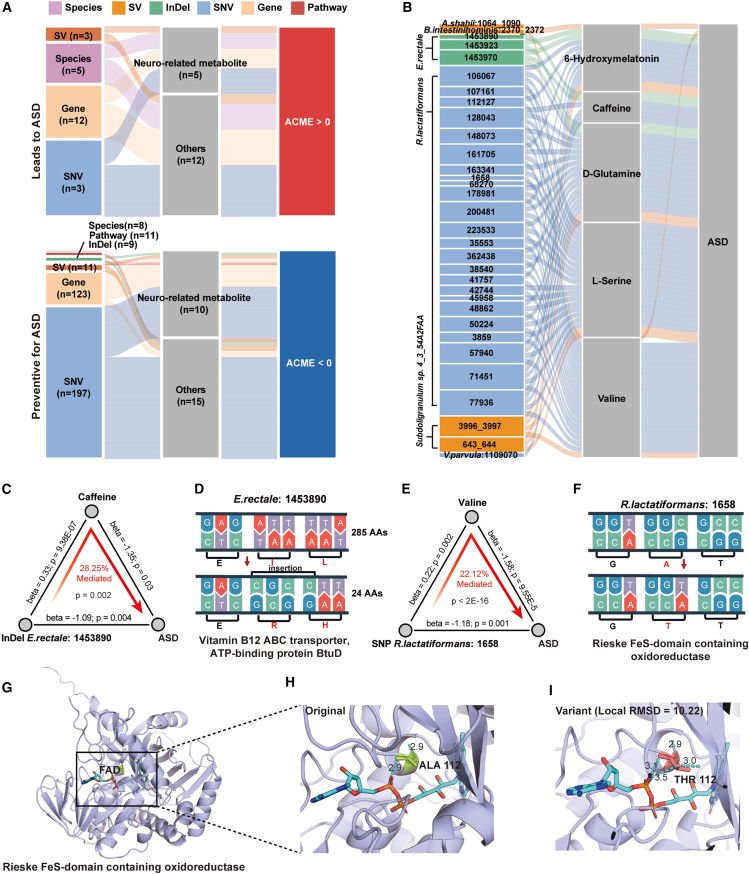
Causal inference of microbiome-metabolite interactions via mediation analysis (A) Sankey diagram illustrating the inferred mediation linkages across multi-dimensional data types, based on mediation analysis. ACME, average causal mediation effects. (B) Sankey diagram highlighting mediation linkages involving 32 missense variants located within coding sequences (CDS), with neuro-related metabolites identified as mediators. (C and E) Examples of mediation linkages between genomic variants and metabolites inferred by mediation analysis. Arrows denote the direction of effects, with annotated beta coefficients and statistical significance. The proportion of indirect (mediated) effects is indicated at the center. (D and F) Genomic context of the variants shown in (C and E), displaying both wild-type (up panel) and mutated (down panel) nucleotide sequences, along with corresponding amino acid translations. (G) Protein structure of Rieske-type oxidoreductase bound to FAD. (H) Wild-type structure highlighting Ala112 (green), the residue of interest. (I) Mutant structure showing the A112T substitution (Thr in pink) and its newly formed hydrogen bond with FAD (blue dashed line). See also Data S9.

To investigate whether the observed associations between microbial features and metabolites contribute to ASD-related neurological phenotypes, we next performed mediation analysis. Starting from 529 significantly associated microbial features and 77 metabolites, this approach identified 1,977 significant mediation relationships (FDR <0.05), spanning 13 microbial species, 14 SVs, 9 InDels, 200 SNVs, 135 microbial genes, 11 functional pathways, and 28 metabolites (Figure 5A). Notably, 11 of the mediating metabolites were neuro-related, suggesting their potential roles in linking gut microbial activity to host neurophysiology. (Figure 5A; Data S9).

Functional annotation revealed 32 protein-altering variants across six species that were involved in 83 neuro-related metabolite-mediated associations (Figure 5B). One representative example is a 4-bp insertion in *Eubacterium rectale* that results in premature truncation of BtuD, a vitamin B12 ABC transporter, reducing the encoded protein from 285 to 24 amino acids. Given the essential role of vitamin B12 in both microbial and host neurotransmitter synthesis, a variant that impairs B12 uptake could plausibly affect neurodevelopment via methylation-dependent pathways and increase ASD risk, potentially mediated by altered caffeine metabolism. (Figures 5C and 5D). Another representative variant is a non-synonymous SNV at position 1650 in *R. lactatiformans*, located within the gene encoding Rieske-type oxidoreductase. This variant was significantly associated with ASD through valine as a putative mediator (Figure 5E). It results in an Ala→Thr substitution within the flavin adenine dinucleotide (FAD)-binding domain of Rieske-type oxidoreductase (Figure 5F), a critical region for enzymatic redox activity. Structural modeling revealed a marked conformational rearrangement in the FAD-binding pocket (local root-mean-square deviation = 10.22 Å), along with an increased number of hydrogen bonds between the FAD cofactor and surrounding residues (from 2 to 4), potentially altering binding stability and redox activity (Figures 5G–5I).

### Multi-level integration of metagenomic signatures for ASD diagnosis

**Figure 6:**
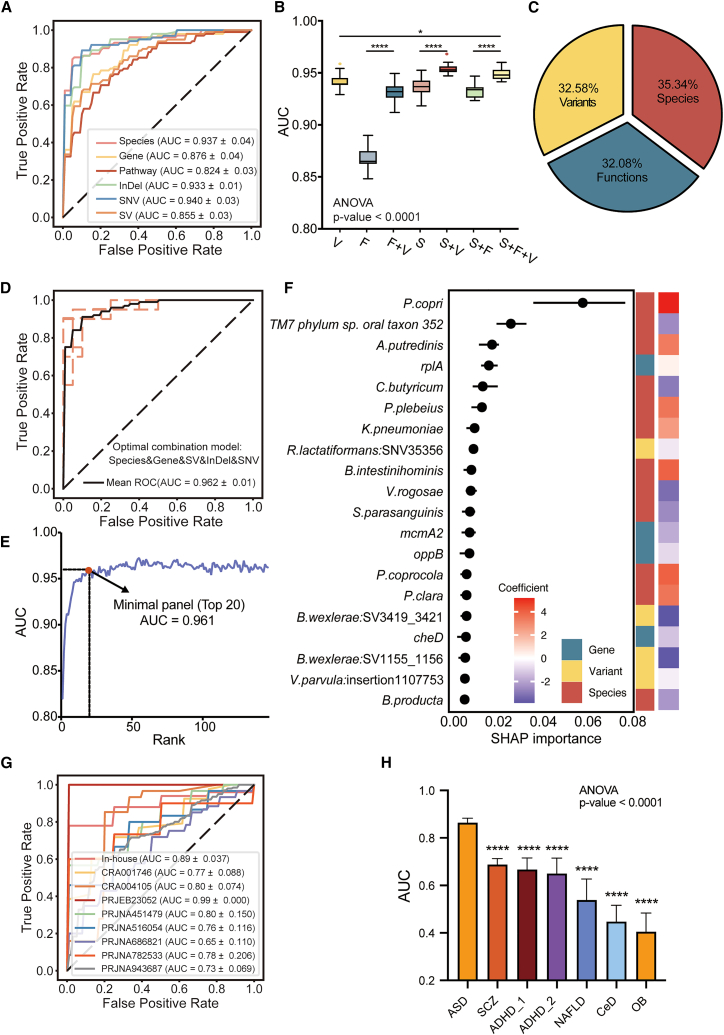
The diagnostic models of ASD based on microbial multi-dimensional biomarkers (A) The receiver operating characteristic curves from 5-fold cross-validation of the species, gene, pathway, SV, InDel, and SNV diagnostic models. (B) Comparison of model performance using species, function, or variant features alone, as well as combinations of feature types. *p* values were calculated using ANOVA with Tukey’s post-hoc test. S, species; F, microbial genes and pathways; V, genomic variants. (C) Relative contribution of each feature type in the integrated model (species, functions, SNVs, InDels, and SVs), calculated using group-normalized SHAP values, where the summed contribution of each feature class was normalized by the number of features in that class. (D) The ROC curves from 5-fold cross-validation of the optimal combination model. (E) AUC curve depicting performance changes as features were incrementally added to the model according to descending importance rankings. (F) Feature importance of the 20-marker minimal panel. Feature types and their direction of change in ASD are indicated. (G) External validation of the 20-microbial marker model across nine independent ASD cohorts, showing AUC values per cohort. (H) Specificity assessment of the 20-marker across non-ASD disease diseases: ADHD (two cohorts), schizophrenia (SCZ), NAFLD, celiac disease (CeD), and obesity (OB). *p* values were calculated using ANOVA with Tukey’s post-hoc test, comparing each non-ASD disease group with ASD (∗*p* < 0.05, ∗∗∗∗*p* < 0.001). See also Data S10 and S11.

To assess the diagnostic utility of gut microbiome alterations in ASD, we developed classification models integrating features across five layers: taxonomic, functional, and genomic variation (SVs, InDels, SNVs). A feature selection pipeline (Triple-E from xMarkerFinder^40^) identified a set of 154 non-redundant microbial markers, including 19 species, 16 genes, 8 pathways, 62 SVs, 35 InDels, and 14 SNVs (Data S10). Among individual feature types, SNVs achieved the highest accuracy (area under the receiver operating characteristic curve [AUC] = 0.940), with species (AUC = 0.937), InDels (AUC = 0.933), genes (AUC = 0.876), SVs (AUC = 0.855), and pathways (AUC = 0.824) following (Figure 6A). Notably, integrating variant-level features with species- or function-level features significantly improved model performance (Figure 6B). Similarly, combining species and functional features likewise improved the predictive power of the variants-only model (Figure 6B). Furthermore, a group-normalized Shapley Additive Explanations (SHAP) analysis showed that the relative contribution of each major feature class was highly balanced, with species contributing 35.34%, variants 32.58%, and functions (genes and pathways) 32.08% (Figure 6C). Together, these results demonstrate that the diagnostic gains are driven by the complementary and synergistic contributions of multi-level features.

The model integrating five microbial layers—species, gene, SV, InDel, and SNV—achieved the best overall classification performance (AUC = 0.962) (Figure 6D), with consistently high values across eight performance indicators, including Matthews correlation coefficient = 0.840, sensitivity = 0.911, specificity = 0.929, and overall accuracy = 0.920 (Data S11). To define a minimal yet robust diagnostic signature, we ranked features by importance and incrementally introduced them into the model. A compact panel of 20 microbial features achieved nearly equivalent accuracy (AUC = 0.961) (Figures 6E and 6F), including 13 species, 3 genes, and 4 genomic variants. Within this panel, *P. copri* ranked highest in importance, consistent with its pervasive alterations across taxonomic, functional, and genetic levels in ASD (Figure 3B). Other ASD-enriched taxa included *Phocaeicola plebeius*, *A. putredinis*, *Barnesiella intestinihominis,* and *P. coprocola*. In contrast, *C. butyricum*, *Veillonella rogosae*, *Streptococcus parasanguinis*, and *Blautia producta* were depleted in ASD. Functionally, the panel included *rplA* (K02863, ribosomal gene), *mcmA2* (K01849, encoding the large subunit of methylmalonyl-CoA mutase involved in SCFA metabolism, also contained differential SNVs), and *oppB* (part of the oligopeptide transport system). Genomic variants retained in the panel included an SNV in *R. lactatiformans* (position 35356), an insertion in *V. parvula* (1107753), and two SVs from *B. wexlerae* (3419_3421 kb and 1155_1156 kb).

To externally validate this diagnostic panel and mitigate the risk of overfitting, we applied the 20-feature model to an independent in-house cohort and eight external ASD cohorts from four countries or regions (Figure 1A). Across these external datasets, the model generally achieved robust performance (median AUC = 0.78; range 0.65–0.99) (Figure 6G). Notably, high-ranking features, such as *P. copr*i (ranked first in the discovery cohort) and *rplA* (ranked fourth in the discovery cohort), appeared among the top five features in 7 of 10 cohorts, demonstrating consistent importance across validation cohorts (Figures S6A and S6B). More importantly, it showed comparable performance for males and females, confirming the applicability of our model across genders (Figure S8). To further ensure a low false-positive rate for the diagnosis of ASD, we applied the panel to five non-ASD conditions, including neurodevelopmental/psychiatric disorders (ADHD and SCZ) and pediatric dysbiosis-associated diseases (CeD, NAFLD, and OB). The panel demonstrated high diagnostic specificity, with significantly lower AUCs and distinct microbial signatures in non-ASD diseases compared to ASD (Figures 6H and S6C).

### Integration of gut microbiome and metabolome improves diagnostic performance for ASD

To further improve diagnostic accuracy, we incorporated fecal metabolomic signatures into the microbiome-based model. At the metabolite level, 28 diagnostic markers were identified using the same feature selection framework, yielding a standalone metabolomics-based model with an AUC of 0.97 (Data S10).

When combining multi-omics features, the integrated model incorporating species, microbial functions, genomic variants, and metabolomic markers achieved the highest diagnostic performance (AUC = 0.98) (Figure S7A). Feature contribution analysis using feature importance indicated that metabolomic features contributed the largest proportion to the integrated model, underscoring the critical value of metabolic signals (Figure S7B).

To derive a parsimonious yet accurate model, we selected the top 20 features from the full model. This minimal panel achieved an AUC of 0.99 in the training cohort, with contributions spanning 6 species (e.g., *P. copri*, *A. putredinis*, and *P. plebeius*), 1 microbial gene (*rplA*, K02863), 1 pathway (tetrapyrrole biosynthesis I from.glutamate), 1 SNV in *R. lactatiformans*, and 11 metabolites including hexadecanamide, L-glutamic acid, indole-3-carboxylic acid, and 2-oxoglutaric acid (Figure S7C).

Importantly, external validation using the independent in-house dataset confirmed the robustness of the model. The 20-marker multi-omics panel (microbiome + metabolome) achieved an AUC of 0.90, slightly higher than that of the microbiome-only model using the same number of markers (AUC = 0.890), demonstrating added value of metabolomic features (Figure S7D).

Taken together, these results highlight the diagnostic utility of a refined multi-dimensional marker panel combining microbial species, functions, variants, and host metabolites.

## Discussion

### Genomic variation uncovers hidden microbial and functional alterations in ASD

Prior studies on ASD have primarily focused on taxonomic and gene-level shifts in the gut microbiome,^14^^,^^15^^,^^19^^,^^20^ overlooking genetic variations. In this study, we performed a comprehensive multi-layered analysis of the gut microbiome—including species-level composition, microbial gene and pathway profiles, and genomic variants (SVs, InDels, and SNVs), as well as untargeted fecal metabolomics, with data from over 1,100 children across 10 cohorts. Our findings demonstrate that ASD is associated with pervasive alterations not only in microbial taxa and function but also at the genomic variant levels. Crucially, variant-level profiling uncovered functional alterations that were either partially aligned with or entirely independent of traditional taxonomic findings, highlighting its potential to capture hidden microdiversity relevant to ASD pathogenesis.

We first confirmed known ASD-associated taxonomic patterns: several enriched species—including *P*. *copri,*^35^ *K. pneumoniae,*^36^ *Parabacteroides merdae,*^12^ and *A. putredinis,*^35^ have been previously linked to neuroinflammation, compromised epithelial barrier function, or microbial-host immune interactions. Conversely, multiple SCFA-producing taxa, especially within the Clostridia class, were depleted in ASD, consistent with prior evidence of metabolic dysregulation.^32^^,^^41^ Notably, *P. copri* stood out by exhibiting consistent alterations across all three types of genomic variants (SVs, InDels, SNVs), underscoring its central role in ASD-associated microbial shifts.

Beyond these well-characterized taxa, our variant-level analysis also uncovered alterations from species not differentially abundant at the compositional level. For example, *B. wexlerae*, *E. rectale*, and *Subdoligranulum* sp. *4_3_54A2FAA* harbored a high burden of ASD-associated genomic variants. Although rarely highlighted in the ASD literature, these species have been implicated in neurodevelopmental or immune signaling pathways in other gut microbiome-related conditions.^28^^,^^42^^,^^43^^,^^44^^,^^45^ This suggests that strain-level remodeling, rather than changes in relative abundance, may substantially impact microbial function in ASD.

Supporting this notion, enzyme-level integration highlighted 196 variant-associated enzymes. Notably, the most affected pathways—identified across taxonomic, functional, and genomic layers—were consistently enriched for carbohydrate metabolism, amino acid metabolism, and SCFA biosynthesis, aligning with prior evidence.^46^ In addition, genomic variation uniquely emphasized alterations in nucleotide metabolism, and two-component system, suggesting potential impacts on microbial replication, signaling, and environmental responsiveness.

Although previous studies have reported associations between gut microbiome features and ASD behavioral scores,^47^^,^^48^ we did not identify robust links between microbial genomic variants and standardized clinical assessments. Given the heterogeneity and context dependence of ASD measures, these relationships are likely more complex than case-control status. Larger, deeply phenotyped cohorts may allow evaluation of microbiome-based scores and clarify links between microbial genomic variation and symptom dimensions.

### Variant-metabolite interactions implied microbial drivers of dysregulated host-microbiome crosstalk in ASD

Beyond taxonomic and functional shifts, our metabolomics analysis revealed widespread alterations in fecal metabolites among children with ASD. Significant reductions were observed in glutamatergic and GABAergic metabolites^49^ (e.g., L-glutamic acid, L-aspartic acid, and N-acetylaspartylglutamic acid), monoamines^50^ (dopamine, 6-hydroxymelatonin, and caffeine), and tryptophan derivatives^38^ (N-methyltryptamine and indole-3-carboxylic acid). Notably, many of these metabolites exhibited strong associations with specific microbial taxa and genetic variants, suggesting interconnected microbial-metabolic dysregulation in ASD.

Building on prior work demonstrating that microbial metabolites can modulate host physiology,^23^^,^^24^ we applied mediation analysis to explore the potential mediating roles of metabolites between microbes and ASD. This analysis identified 28 metabolites that putatively mediated 382 associations between microbial features and ASD status. Of these, 11 were neuro-related compounds and 223 were microbial variant features, including 32 predicted to alter protein-coding sequences. Two examples further illustrate potential mechanistic pathways. An InDel in the *BtuD* gene (vitamin B12 ABC transporter) resulted in a truncating mutation associated with elevated fecal caffeine levels and reduced ASD risk via indirect mediation. Given caffeine’s reported neuroprotective effects,^51^ this suggests that disrupted B12 uptake may alter microbial degradation or transformation of neuroactive compounds.^52^ Another example involved an SNV in a Rieske-type oxidoreductase, predicted to impair FAD cofactor binding and redox efficiency. This class of enzymes typically participates in electron transfer reactions and the oxidative metabolism of aromatic or xenobiotic compounds, including those derived from amino acid or neurotransmitter catabolism.^53^^,^^54^ Altogether, our mediation analyses suggest potential mediating roles of specific metabolites and pathways through which gut microbial variation may influence ASD, offering functional hypotheses that warrant further investigation and validation in future studies. However, there are still a majority of ASD-associated variants that are located in non-coding regions or do not alter protein sequences. Future studies integrating transcriptomic or proteomic data are needed to clarify the functional impact of these variants.

The present study focused on stool metabolomics, chosen for its proximity to microbial activity, sensitivity to gut-derived metabolites, and suitability for non-invasive, longitudinal sampling. Although plasma metabolomics data were not available in the present study, prior studies have reported similar metabolic changes in ASD serum, including glutamate^46^ and tryptophan derivatives,^55^ which were also detected in our data. Moving forward, integrating fecal, plasma, and other biospecimen data will be essential to establish a more comprehensive and persuasive multi-site view of ASD-associated metabolic dysregulation.

### A multi-layered microbial and metabolomic signature enables robust, non-invasive ASD diagnosis

Finally, by integrating taxonomic, functional, and genomic variation features into a unified classification framework, we developed a robust diagnostic model for ASD. Using machine learning with stringent feature selection, we identified a compact yet high-performing 20-marker panel comprising 13 species, 3 microbial genes, and 4 genomic variants. Notably, *P. copri* ranked highest in predictive importance, consistent with its multi-level alterations in ASD. This 20-marker microbiome panel achieved high accuracy in the primary cohort (AUC = 0.961) and demonstrated robust performance across 9 external cohorts (median AUC = 0.78), despite the existence of inter-study variability, outperforming all previous non-invasive diagnostic models.^14^^,^^20^ Notably, the relatively lower AUC observed in PRJNA686821 may partly reflect diagnostic heterogeneity (Diagnostic and Statistical Manual of Mental Disorders [DSM]-IV/DSM-V), whereas other DSM-V-based cohorts showed higher transferability and accuracy. Importantly, it also demonstrated strong disease specificity, with substantially lower AUCs observed in other pediatric dysbiosis-associated conditions as well as in neurodevelopmental and psychiatric disorders. Together, these results underscore that taxonomic composition, functional potential, and genomic variation each provide complementary and synergistic contributions that are indispensable for a comprehensive understanding of the gut microbiome’s role in ASD. Furthermore, the inclusion of untargeted metabolomic data further enhanced diagnostic performance, yielding a 20-marker multi-omics model that achieved an AUC of 0.99 in internal validation and 0.90 in an independent multi-center validation cohort. While the inclusion of metabolomic data provides notable performance gains; the microbiome-only 20-marker panel offers greater adaptability and scalability for broader diagnostic use.

Biologically, several panel features align with established gut-brain pathways: (1) microbial tryptophan/indole metabolism^56^ (e.g., indole-3-carboxylic acid), (2) SCFA pathways^41^ involving *mcmA2* (methylmalonyl-CoA mutase) and SCFA-associated taxa (*C. butyricum*, *F. plautii*, *Anaerotruncus colihominis*), and (3) microbial GABA/glutamate metabolism^49^ reflected by reduced L-glutamic acid. These literature-anchored pathways provide a biologically coherent framework for the observed ASD specificity and offer testable hypotheses for future causal studies (e.g., pathway-level perturbation, gnotobiotic transfer, and metabolite supplementation).

A prevailing concern in ASD microbiome research is whether observed microbial features are truly disease related or merely reflect dietary preferences commonly seen in children with ASD.^57^^,^^58^^,^^59^ Notably, taxa such as *P. copri* are diet- responsive,^60^ and prior studies have shown that dietary influences can shape microbial genomic structure, including SVs.^25^ While such effect cannot be entirely excluded, the robust generalization of our model across cohorts with diverse geographic and dietary backgrounds, combined with its maintained specificity against non-ASD dysbiosis-associated conditions, suggests that diet alone is insufficient to explain the findings.

Our study highlights the potential of multi-level microbial markers for ASD diagnosis, yet several challenges remain before clinical translation. While our model showed good generalizability, the absence of comprehensive dietary information and stratification by gastrointestinal comorbidities limits our ability to fully disentangle disease-specific signals from environmental influences. Looking ahead, larger multi-center cohorts spanning diverse regions, together with standardized cross-cohort integration strategies, will be essential before clinical application. Ideally, these cohorts would include paired metagenomic and metabolomic data as well as comprehensive metadata. Moreover, future work should evaluate model robustness under varying sequencing depths and assess targeted approaches (e.g., qPCR, amplicon-based panels, or droplet digital PCR) as cost-effective, scalable strategies for routine diagnostics.

In conclusion, our study presents a multi-layered characterization of the gut microbiome in ASD, highlighting the added value of variant-level profiling in revealing a previously underexplored dimension of microbiome. Integration with metabolomic data uncovered potential mechanistic links between microbial mutations and host neurochemical imbalance. Finally, we developed a compact and generalizable diagnostic model, further enhanced by metabolomic features, offering a promising foundation for early, non-invasive ASD detection and microbiome-targeted interventions.

### Limitations of the study

Our study has several limitations. First, dietary factors and gastrointestinal comorbidities remain potential confounders. Therefore, comprehensive metadata will be needed to confirm the links we identified between microbial features and ASD. Second, although we employed cross-cohort validations, most cohorts were from high-income regions and several cohorts had relatively small sample sizes. Larger cohorts with broader geographic and ethnic representation are needed to ensure global generalizability. In addition, external validation cohorts with paired metagenomic and metabolomic data will be needed to further confirm the robustness of metabolomic biomarkers. Last, as this is a cross-sectional study, we inferred putative mediating relationships between microbial signatures, metabolites, and ASD phenotypes using mediation analysis. However, these putative mediating relationships still require confirmation through longitudinal study designs and experimental validation. Future work should incorporate more rigorous matching strategies (e.g., age-matched siblings or twins)^61^^,^^62^ and apply absolute quantification^63^^,^^64^ to better resolve host-microbe interactions.

## Resource availability

### Lead contact

For additional information and inquiries regarding resources, kindly direct your correspondence to the lead contact, Lixin Zhu (lixinzhu@imu.edu.cn).

### Materials availability

This study did not generate any novel or unique reagents.

### Data and code availability

• The in-house metagenomic sequencing data generated in both the discovery and validation cohorts have been deposited in the National Omics Data Encyclopedia (NODE; https://www.biosino.org/node/) under NODE: OEP00004172 and NODE: OEP00006635. The corresponding untargeted metabolomics data are available under NODE: OEP00004197 at the same repository. Additional metagenomic datasets used in this study are accessible from multiple repositories. Data from the Genome Sequence Archive (GSA; https://ngdc.cncb.ac.cn/gsa/) can be found under GSA: CRA001746, GSA: CRA004105; data from the China National GeneBank DataBase (CNGB; https://db.cngb.org/) can be found under CNGB: CNP0000119, CNGB: CNP0000729; and additional data from NODE are available under NODE: OEP00006634. Other datasets are accessible via the NCBI Sequence Read Archive under the following BioProject identifiers: SRA: PRJNA516054, SRA: PRJEB23052, SRA: PRJNA782533, SRA: PRJNA451479, SRA: PRJNA686821, SRA: PRJNA943687, and SRA: PRJNA759642.
• All original code has been deposited at GitHub (https://github.com/tjcadd2020/ASD-microbiome_metabolomics) and is publicly available as of the date of publication.
• Any additional information required to reanalyze the data reported in this work paper is available from the lead contact upon request.

## Acknowledgments

The authors would like to thank all the researchers for generously sharing their sequencing data included in this study. We acknowledge funding from the 10.13039/501100001809National Natural Science Foundation of China (grant numbers 92251307 and 82170542 to R.Z., 32470098 to N.J., and 82300753 to X.W.), the National Key R&D Program (grant number 2024YFA1307100/2024YFA1307101 to H.Q.), the Projects of the Central Government in Guidance of Local Science and Technology Development (grant number YDZX20213100003690 to H.Q.), the 10.13039/501100014137Shanghai Shenkang Hospital Development Center Project (grant number SHDC2020CR1030B to H.Q.), top priority project of Shanghai (grant number 076478684Q/2023-00154 to H.Q.), and the Doctoral Student Special Project of the Young Talent Support Program by the 10.13039/100010097China Association for Science and Technology (CAST) (to W.C.). The funders had no role in study design, data collection and analysis, decision to publish, or preparation of the manuscript. We acknowledge the computational support provided by the Center for Scientific Computing and the 10.13039/501100021520Supercomputing Center of the School of Life Sciences and Technology, 10.13039/501100004204Tongji University.

## Author contributions

L. Zhu, R.Z., H.Q., and J.W. conceived and designed the study. W.C. W.G., L.T., X.Z., W.Y., and N.J. performed public data collection and bioinformatics analysis. R.Y., Y.G., H.Z., and L.H. were responsible for the clinical diagnosis and enrollment of autism patients, while Q.W. and Y.Z. were responsible for the clinical diagnosis and enrollment of typically developing healthy children. J.W., R.Y., Y.G., H.Z., and L.H. jointly collected the fecal samples required for the discovery cohort. X.W., L. Zhang, L.W., G.Y., and Y.L. were responsible for collecting the fecal samples for the validation cohort. R.Z., H.Q., N.J., and X.W. provided the research funding support required for this project. W.C. wrote the first draft. All other authors critically revised the manuscript, and all authors reviewed and approved the final version before submission.

## Declaration of interests

The authors declare no competing interests.

## STAR★Methods

### Key resources table

**Table:** 

REAGENT or RESOURCE	SOURCE	IDENTIFIER
**Biological samples**		

Human feces	Shanghai Tenth People’s Hospital, Suzhou Municipal Hospital	N/A

**Deposited data**		

Raw metagenomic data	This paper, Li et al.,^65^ Wan et al.^66^	NODE: OEP00004172, NODE: OEP00006635, CNGB: CNP0000729, NODE: OEP00006634
Metabolomic data	This paper	NODE: OEP00004197
Publically available dataset	Zhang et al.,^48^ Tong et al.,^67^ Wang et al.,^33^ Dan et al.,^68^ Kovtun et al.,^69^ Wan et al.,^15^ Nirmalkar et al.,^18^ Su et al.,^14^ Mouzan et al.,^70^ Testerman et al.,^71^ Murga-Garrido et al.,^72^ Zhu et al.^73^	GSA: CRA001746, GSA: CRA004105, SRA: PRJNA516054, SRA: PRJEB23052, SRA: PRJNA782533, SRA: PRJNA451479, SRA: PRJNA686821, SRA: PRJNA943687, SRA: PRJNA759642, SRA: PRJNA398089, SRA: PRJNA757365, SRA: PRJNA328258, SRA: PRJNA721692, CNGB: CNP0000119

**Software and algorithms**		

xMarkerFinder	Gao et al.^40^	https://github.com/tjcadd2020/xMarkerFinder
MaAsLin2	Mallick et al.^74^	https://github.com/biobakery/Maaslin2
KneadData v.0.6	http://huttenhower.sph.harvard.edu/kneaddata	https://github.com/biobakery/kneaddata
MetaPhlAn4	Blanco-Miguez et al.^75^	http://huttenhower.sph.harvard.edu/metaphlan
HUMAnN3	Beghini et al.^76^	https://github.com/biobakery/humann
SGVFinder	Zeevi et al.^25^	https://github.com/segalab/SGVFinder
GATK HaplotypeCaller	DePristo et al.^77^	https://gatk.broadinstitute.org/hc/en-us
mediation	Tingley et al.^78^	https://cran.r-project.org/package=mediation
AlphaFold3	Abramson et al.^79^	https://alphafoldserver.com/
Fpocket	Guilloux et al.^80^	https://github.com/Discngine/fpocket
PyMOL	Schrödinger, Inc.	https://www.pymol.org/

### Experimental model and study participant details

#### Participants of the discovery cohort

Participants with ASD and neurotypical controls were enrolled at the Shanghai Tenth People’s Hospital between February 2021 and February 2023. Children diagnosed with ASD based on the Diagnostic and Statistical Manual of Mental Disorders, Fifth Edition (DSM-5) criteria and presenting with recurrent gastrointestinal symptoms (including diarrhea, constipation, abdominal distension, or food allergy/intolerance for ≥6 months and within the past 3 months) were enrolled. ASD diagnoses were further confirmed using the Childhood Autism Rating Scale (CARS) and the Autism Behavior Checklist (ABC). For GI symptoms, Gastrointestinal Symptom Rating Scale (GSRS) was used. Age- and gender-matched neurotypical children without gastrointestinal symptoms were recruited as controls from local public schools. All participants underwent standardized neurological, physical, and behavioral evaluations conducted by board-certified pediatric psychiatrists in the Department of Pediatrics at the same institution.

Exclusion criteria for all participants included: known medical or neurological conditions; diagnosis of organic gastrointestinal disease; severe malnutrition or obesity; use of antibiotics, probiotics, prebiotics or other microbiota-influencing medications within the past 3 months; or receipt of fecal microbiota transplantation within the past 12 months.

In total, 102 children with ASD and 99 neurotypical controls of Han Chinese ethnicity, aged 3 to 10 years, were enrolled. All the parents of those participants provided written informed consent to participate in the study, which was approved by the Ethics Committee of the Tenth People’s Hospital, Tongji University (Approval No. SHSY-IEC-5.0/21k8/P05 and K-2025-093-K01).

#### Participants of the validation cohorts

##### Independent in-house ASD cohort

To independently validate the selected microbial and metabolic markers as well as trained classification models, we recruited an independent hospital-based cohort between 2021 and 2025 from Shanghai Tenth People’s Hospital (Tongji University) and Suzhou Municipal Hospital (Nanjing Medical University). This cohort comprised 69 children (ASD: 49; TD: 20), and all fecal samples were subjected to the same metagenomic and untargeted metabolomic profiling procedures as in the discovery cohort. Inclusion and exclusion criteria were consistent with those applied in the discovery cohort; ASD participants additionally required a confirmed clinical diagnosis and a pre-sampling dietary record. To avoid participant overlap, enrollment records and clinical identifiers were cross-checked to ensure that all individuals were unique to this cohort. The study protocol was reviewed and approved by the Ethics Committee of Suzhou Municipal Hospital, Suzhou (Approval ID: K-2025-093-K01).

##### Independent in-house ADHD cohorts

To further evaluate the specificity of ASD-associated microbial signatures, we incorporated two independent in-house ADHD cohorts. The first cohort comprised 25 children with ADHD and 25 age-matched neurotypical controls recruited from the Pediatric Outpatient Department of the First Medical Center of the PLA General Hospital between January and June 2019. This study was approved by the Ethics Committee of the PLA General Hospital (Approval ID: 2018-278). The second cohort included 98 children and adolescents with ADHD and 109 neurotypical controls originally recruited at Xijing Hospital, Shaanxi, China, between March 2018 and February 2020; for the present study, we restricted the analysis to children aged ≤8 years to achieve closer age matching with our ASD cohorts, yielding a total of 35 ADHD cases and 43 neurotypical controls. For both cohorts, stool samples were collected under standardized protocols, and detailed recruitment procedures, diagnostic assessments, and exclusion criteria have been described previously in the original publications.^65^^,^^66^ This study was approved by the Ethics Committee of Xijing Hospital, Fourth Military Medical University (Approval ID: KY20182002-1) and was also registered at ClinicalTrials.gov (Identifier: NCT03447223).

#### Public validation cohorts

To extend the validation across different populations and geographic regions, we compiled publicly available whole metagenome shotgun sequencing datasets from eight previously published ASD studies, covering four countries or regions. These included the ZhangM 2020^48^ cohort (ASD: 39; TD: 40, GSA: CRA001746), TongZ 2022^67^ (ASD: 26; TD: 26, GSA: CRA004105), WangM 2019^33^ (ASD: 43; TD: 31, SRA: PRJEB23052), DanZ 2020^68^ (ASD: 30; TD: 30, SRA: PRJNA451479), KovtunAZ 2020^69^ (ASD: 30; TD: 20, SRA: PRJNA516054), WanY 2021^15^ (ASD: 63; TD: 59, SRA: PRJNA686821), NirmalkarK 2022^18^ (ASD: 18; TD: 20, SRA: PRJNA782533), and SuQ 2024^14^ (ASD: 201; TD: 177, SRA: PRJNA943687).

To evaluate the specificity of ASD-associated microbial signatures, we incorporated not only in-house ADHD cohorts but also publicly available datasets from other psychiatric disorders, such as schizophrenia (schizophrenia^70^: 90; control: 81, CNGB: CNP0000119), as well as pediatric cohorts of other microbiota-associated diseases, including celiac disease (CeD^71^: 19; control: 20, SRA: PRJNA757365), non-alcoholic fatty liver disease (NAFLD^72^: 12; control: 12, SRA: PRJNA328258), and obesity (OB^73^: 17; control: 23, SRA: PRJNA721692). All raw metagenomic sequencing data were downloaded from the National Genomics Data Center (NGDC), China National GeneBank database (CNGBdb) and the European Nucleotide Archive (ENA).

### Method details

#### Stool sample collection

Fecal samples were collected from all participants within 30 min of defecation. To prevent contamination with urine, feces were collected directly into a designated container. Approximately 20 grams of each sample were obtained using a Shaster sampling tube and divided into 2 mL cryotubes, with each tube containing 1 gram of sample. The samples were immediately flash-frozen in liquid nitrogen and stored at −80°C for cryopreservation. Microbiome analysis was conducted within six months.

#### Metagenomic sequencing data processing

Genomic DNA was extracted from fecal samples using a stool DNA Kit (QIAamp Power Fecal Pro DNA Kit), following the manufacturer’s instructions. After the quality control, extracted DNA was subjected to metagenomic libraries construction by Hieff NGS OnePot II DNA Library Prep Kit for Illumina (Yeasen, Shanghai, China). Then, high-throughput sequencing was performed on the Novaseq 6000 platform (Illumina, Inc., San Diego, CA, USA) platform.

Raw metagenomic sequencing reads were processed using KneadData (v0.6; http://huttenhower.sph.harvard.edu/kneaddata) to remove low-quality reads and potential contaminants. Quality trimming was performed using Trimmomatic (v0.39), integrated within KneadData, with the following parameters: SLIDINGWINDOW:4:20 MINLEN:50 LEADING:3 TRAILING:3. Host-derived reads and other unwanted sequences—including those mapping to the human genome, bacterial plasmids, UniVec contaminants, and chimeric sequences—were removed using Bowtie2^74^ (v2.4.1) as implemented in KneadData. The final dataset retained a median of 92.13 million high-quality paired-end reads per sample (IQR: 81–100 million) after quality control and contaminant removal (Data S12).

#### Microbial taxonomic and functional profiles

Microbial taxonomic profiles were generated using MetaPhlAn4^75^ (http://huttenhower.sph.harvard.edu/metaphlan), which utilizes clade-specific marker genes to estimate relative abundances of microbial taxa. Profiles were normalized to relative abundance prior to downstream analysis. Microbial functional profiles were obtained using HUMAnN3^76^ (v3.7; https://huttenhower.sph.harvard.edu/humann). Gene abundances and pathway abundances were quantified and converted to relative abundance. For both taxonomic and functional features, only those detected in more than 10% of the samples were retained for subsequent statistical analysis.

#### Microbial genomic variants detection and annotation

For microbial SV analysis, we followed the original workflow described by Zeevi et al.,^25^ using SGVFinder (v1.0) with default parameters. Briefly, the procedure involves: (i) resolving ambiguous multi-mapping reads via the iterative coverage-based read assignment (ICRA) algorithm, which reassigns them to the most likely reference according to mapping quality and genomic coverage; and (ii) splitting reference genomes into bins and identifying highly variable segments based on coverage profiles across samples to detect SVs. Two types of SVs were identified: deletion SVs (dSVs), defined as genomic regions with no coverage in 25–75% of samples, and variable SVs (vSVs), characterized by highly heterogeneous coverage across the cohort. SVs were detected using the default reference database provided by SGVFinder, which is derived from the proGenomes^77^ database (http://progenomes1.embl.de/). In cases where the same region was classified as both a dSV and a vSV, we retained the dSV annotation. SVs present in more than 10% of samples were included in downstream analyses.

For small-scale genomic variation analysis, including insertions/deletions (InDels) and single nucleotide variants (SNVs), we used GATK HaplotypeCaller^78^ (v4.4.0.0) with a customized microbial reference genome set. Due to the generally low prevalence and sparsity of InDels and SNVs in metagenomic data, we limited the analysis to species with high prevalence and abundance (mean relative abundance >0.5% and prevalence >50% across samples) to ensure robust variant calling. A total of 42 reference strains meeting these criteria were selected, as detailed in Data S4. Quality-filtered metagenomic reads were aligned to these reference genomes using BWA-MEM^79^ (v0.7.17). The resulting alignments were sorted with SAMtools^80^ (v1.18), and PCR/optical duplicates were removed using Picard MarkDuplicates (v3.1.0) to minimize potential biases. Variant calling was then performed with GATK HaplotypeCaller (v4.4.0.0) with ploidy set to 1. To ensure robustness, we applied stringent GATK filters (variant calling error probability <1%) and further retained InDels with a sample prevalence >20% and SNVs with a prevalence >40% for downstream analysis.

#### Fecal metabolomics profiling by untargeted LC-MS

Untargeted metabolomic profiling of fecal samples was performed using LC-MS. Briefly, 50 mg of fecal material was extracted in 800 μL of 80% methanol, followed by vortexing, ultrasonication (30 min, 4°C), and centrifugation (12,000 rpm, 15 min, 4°C). The supernatant was collected and spiked with 5 μL of internal standard (2-chlorophenylalanine, 0.14 mg/mL) before LC-MS analysis. Chromatographic separation was conducted on a Waters ACQUITY UPLC HSS T3 column (2.1 × 100 mm, 1.8 μm) using a Waters UPLC system coupled to a Thermo Q Exactive mass spectrometer. The mobile phases were water with 0.05% formic acid (A) and acetonitrile (B). A gradient elution was applied at a flow rate of 0.3 mL/min over 16 min, and 5 μL of each sample was injected. The mass spectrometer operated in both positive and negative ESI modes, using full scan (m/z 70–1050) and data-dependent MS/MS (dd-MS2, Top10), with a resolution of 70,000 (MS1) and 17,500 (MS2).

Raw data were converted to mzML and processed using XCMS for peak detection and alignment. Metabolite annotation was performed using MS-DIAL (v4.9) with public spectral libraries. Features with detection in <20% of samples or with a CV >30% in QC samples were excluded from analysis. QC samples were injected regularly throughout the run to assess signal stability. In total, 652 annotated metabolites were involved in the analysis. These metabolites spanned eight major chemical classes, as defined by the Human Metabolome Database (HMDB).^81^ The characterization of neuro-related metabolites, including glutamine, dopamine, adenosine, valine and acetylaspartylglutamic acid was manually curated based on prior biological knowledge and literature relevance.

### Quantification and statistical analysis

#### Distance matrix-based variance estimation and principal coordinates analysis

To evaluate compositional differences across samples, principal coordinate analysis (PCoA) and ANOSIM were performed based on appropriate distance metrics. Specifically, Bray-Curtis dissimilarity was used to quantify compositional differences in microbial taxonomic profiles, functional profiles (including genes and pathways), and metabolomic profiles. For variant-level features, Jaccard distance was applied to InDel and SNV profiles due to their binary nature. SVs were analyzed separately, with vSVs assessed using Bray-Curtis distance and dSVs using Jaccard distance. The overall dissimilarity for SVs was calculated by averaging the distance matrices derived from vSVs and dSVs.

To determine whether age or gender confounded the microbial and metabolic differences between ASD and TD groups, we performed permutational multivariate analysis of variance (PERMANOVA, 999 permutations) to quantify the proportion of variance in each data layer explained by these variables. All covariates found to have a significant effect on at least one data type were retained and included as adjustment factors in subsequent statistical models.

#### Species-level associations of the gut microbiome with ASD status

To evaluate the association between ASD and species-level genomic variation, we performed PERMANOVA with 999 permutations using the following formula:

(Equation 1) Distancematrixofvariants(orfunctionalprofiles)∼diagnosis+age+gender

For each microbial species, we constructed sample-wise dissimilarity matrices based on genomic variants (e.g., SNVs, InDels, SVs) or species-stratified functional profiles derived from HUMAnN3 (i.e., gene families and pathways). This allowed us to assess whether species-specific genomic or functional variation was significantly associated with ASD diagnosis, while adjusting for age and gender.

#### Identification of altered gut microbial signatures and metabolomic signatures

To identify microbial and metabolomic features significantly associated with ASD while accounting for potential confounding factors, we applied a linear mixed-effects modeling framework. Specifically, each feature’s abundance (or presence/absence) was modeled as a function of ASD diagnosis, with adjustment for age (as a continuous covariate) and gender (as a binary factor), using the following formula:

(Equation 2) feature∼diagnosis+age+gender

Model fitting was performed using the MaAsLin2^82^ package (Microbiome Multivariable Associations with Linear Models 2) in R. MaAsLin2 applies generalized linear and mixed-effect models to identify associations between microbial or metabolic features and clinical variables, while adjusting for fixed covariates and accommodating repeated measures or zero-inflated data distributions when necessary. The model coefficients represent the effect size (beta coefficient) of the variable of interest (e.g., ASD), relative to the specified reference category. For microbial taxa, we further assessed robustness across multiple differential abundance approaches and retained taxa consistently identified by more than one method. The *p*-value were adjusted using the Benjamini–Hochberg (BH) method. A threshold of false discovery rate (FDR) < 0.01 was employed to identify differential taxa, functions and variants, respectively.

#### Calculation of microbiome–metabolome associations

To evaluate the independent contribution of microbial features to fecal metabolite levels, we employed linear mixed-effects models using lm() function in R, and incorporated microbial features at different levels (e.g., species, SVs, InDels, SNVs, pathways, genes) as explanatory variables. The following model was used:

(Equation 3) metabolite∼microbialfeature+diagnosis+age+gender

The significance of associations was evaluated using the BH method to control the FDR, and results with FDR <0.05 were considered statistically significant.

#### Mediation analysis

To investigate whether fecal metabolites mediate the relationship between microbial features and ASD status, we performed mediation analysis using the mediation package^83^ in R. Microbial features at multiple levels (e.g., species, SVs, InDels, SNVs, pathways, genes) were treated as independent variables (X), ASD diagnosis as the outcome variable (Y), and fecal metabolites as potential mediators (M). Average causal mediation effects (ACME), average direct effects (ADE), and total effects were estimated using a quasi-Bayesian Monte Carlo method with 1,000 simulations. Covariates including age and gender were adjusted in both the mediator and outcome models. The *p*-value were adjusted using the BH method. Significant mediation effects were defined as those with an FDR for the ACME below 0.05, with the 95% confidence interval of the ACME not crossing zero, and with a statistically significant association between the microbial feature and the corresponding metabolite.

#### Protein structure-based analysis

We focused on microbial coding-region variants that were significantly associated with ASD via neuroactive metabolite mediation. Variants located within predicted active or cofactor-binding pockets were prioritized. Fpocket^84^ was used to identify ligand-binding pockets and protein structures were predicted using AlphaFold3.^85^ Ligand information was obtained from UniProt annotations of the corresponding protein domain. Variants located within these pockets were visualized using PyMOL to assess their potential structural and functional impact.

#### Diagnostic model training and testing

To identify robust diagnostic biomarkers and construct predictive models for ASD, we used xMarkerFinder,^40^ a multistep machine learning framework for feature selection and classification. Feature selection was performed in three stages: (1) preliminary screening based on discriminative performance (AUC >0.5), (2) removal of highly collinear features (pairwise Spearman correlation R > 0.7), and (3) recursive feature elimination (RFE) to identify the minimal feature subset required to stabilize model performance. Selected features were used to construct classification models based on the random forest (RF) algorithm. Feature importance was evaluated using SHapley Additive exPlanations (SHAP),^86^ and features were ranked accordingly. Features were progressively added to the model in order of decreasing importance, and the smallest feature set that achieved stable and high performance (AUC) was retained as the final diagnostic panel. To account for potential bias introduced by unequal feature group sizes, we additionally calculated group-normalized SHAP values, in which the summed contribution of each feature class was divided by the number of features in that class.

All models were trained using stratified 5-fold cross-validation to minimize overfitting and preserve the original class distribution. Hyperparameter tuning was performed using the BayesianOptimization package^87^ (v1.4.3) to identify the optimal parameter set. The final diagnostic model was trained using the selected biomarker panel and optimized hyperparameters (Data S10).

To assess the robustness and generalizability of the model, we performed external validation by evaluating each independent cohort separately. In addition, panel’s specificity was assessed by applying the trained model to neurodevelopmental/psychiatric disorders (ADHD, schizophrenia) and pediatric datasets from unrelated conditions (celiac disease, obesity, NAFLD). To minimize technical discrepancies, all datasets were processed through a unified bioinformatics pipeline, with taxonomic and functional profiles normalized to relative abundances, and microbial variant features represented as binary presence/absence (for deletion SVs, InDels, and SNVs) or standardized coverage values (for variable SVs).

#### Statistical analysis

To assess differences in microbial community diversity between ASD and TD children, alpha diversity indices such as Shannon index were calculated using the vegan package^88^ in R.

Considering that microbial data are sparse with a nonnormal distribution, relevant statistics on relative abundance were performed using the ggpubr (v.0.6.0) package (https://github.com/kassambara/ggpubr) with non-parametric tests, such as the Wilcoxon rank-sum test and ANOVA with Tukey’s post-hoc test. Multiple hypothesis testing corrections were done using the false discovery rate (FDR) method. UpSet plots were generated using the UpSetR package^89^ to visualize set intersections across feature groups. Sankey diagrams were created using the ggalluvial package^90^ to illustrate mediation relationships across different microbial levels and metabolites.

All statistical analyses were performed in R (version 4.3).

## Supplemental information

Document S1. Figures S1–S8

Data S1. Demographic information across all cohorts, related to Figure 1

Data S2. The differential analysis results on microbial features of ASD and TD via MaAsLin2, related to Figure 1–2

Data S3. The annotation and differential analysis results on microbial SVs, related to Figure 2

Data S4. Information on 42 reference strains of InDel and SNV annotation, related to Figure 2

Data S5. Differential microbial genomic variations annotated to enzyme-coding genes, related to Figure 3

Data S6. Distribution of differential genomic variants across microbial pathways, related to Figure 3

Data S7. The differential analysis results on metabolites of ASD and TD via MaAsLin2, related to Figure 4

Data S8. Significant associations between microbial features and metabolomic signatures, related to Figure 4–5

Data S9. ASD-associated mediation links between microbial variants and neuro-related metabolites, related to Figure 5

Data S10. Biomarkers used in the diagnostic models, related to Figure 6

Data S11. The performance of diagnostic models based on 5-fold cross-validation, related to Figure 6

Data S12. Sequence data quality control and removal of DNA contamination, related to Figure 1

Document S2. Article plus supplemental information
