# Supplementary material for: Integrative multi-omics reveals microbial genomic variants driving altered host-microbe interactions in autism spectrum disorder
Source: Cell Rep Med. 2025 Dec 19;7(1):102516. doi: 10.1016/j.xcrm.2025.102516 (PMC12866134; doi:10.1016/j.xcrm.2025.102516)
Supplement: Document S1. Figures S1–S8 [file mmc1.pdf]

Cell Reports Medicine, Volume *7*

Supplemental information

Integrative multi-omics reveals microbial genomic variants driving altered host-microbe interactions in autism spectrum disorder

Wanning Chen, Xinjun Wang, Ruixin Zhu, Wenxing Gao, Liwen Tao, Rong Yang, Qing Wei, Yiming Zhang, Yujiao Gong, Hui Zhong, Linsheng Huang, Xinyue Zhu, Yuwei Yang, Linjuan Zhang, Lin Wan, Guang Yang, Yan Li, Na Jiao, Jifeng Wang, Huanlong Qin, and Lixin Zhu

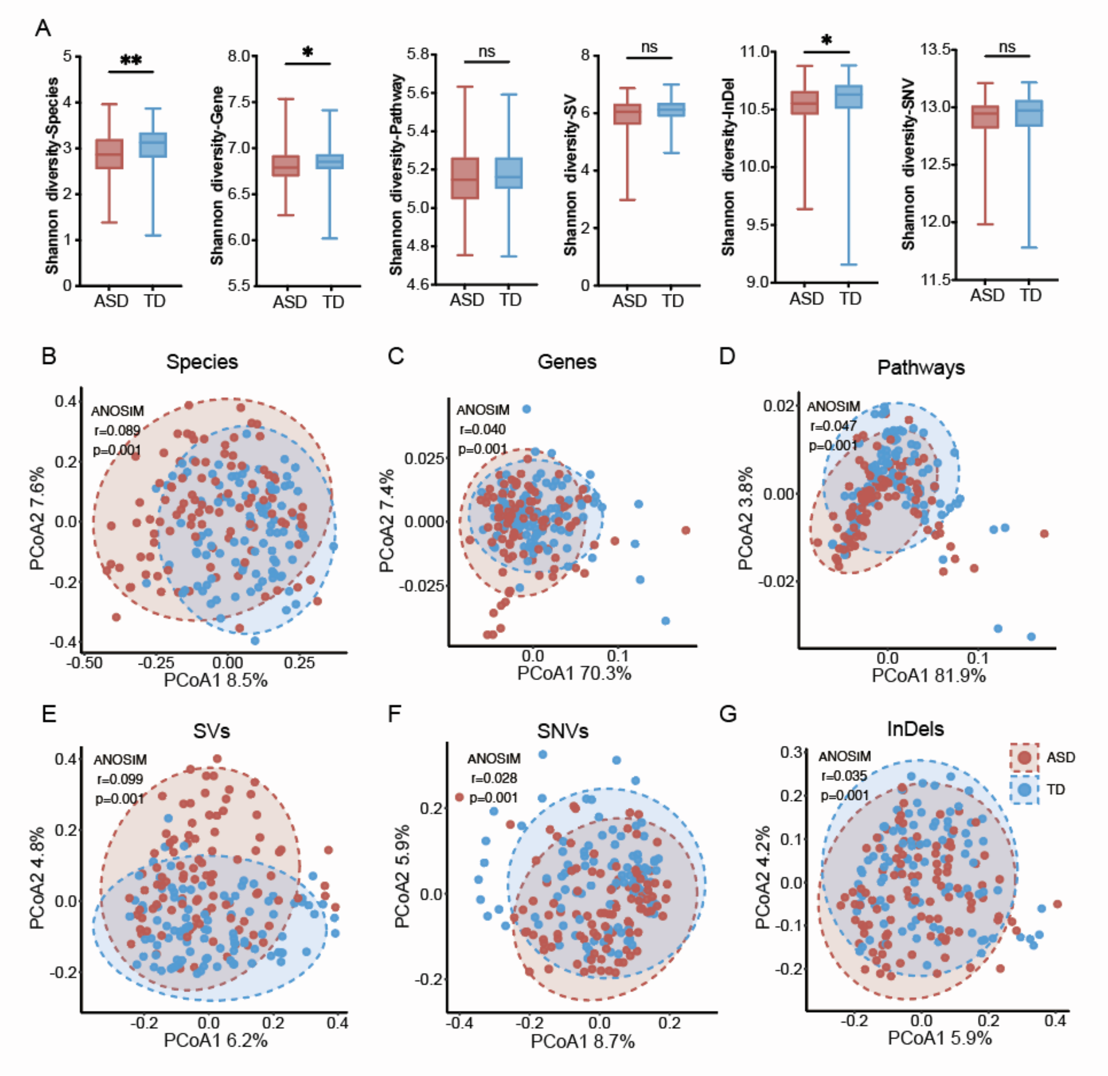

Supplementary figure 1 | Gut microbial ecological diversities in children with ASD and TD. Related to Figure 1-2. (A) Alpha diversity assessed by the Shannon index across ASD and TD groups. Statistical significance was determined using the Wilcoxon rank-sum test (*p < 0.05; **p < 0.01; ns, not significant). (B–G) Principal coordinates analysis (PCoA) based on different microbial feature matrices (species, gene, pathway, single nucleotide variant (SNV), insertion/deletion (InDel), and structural variant (SV) profiles) reveals significant compositional differences between ASD and TD groups.

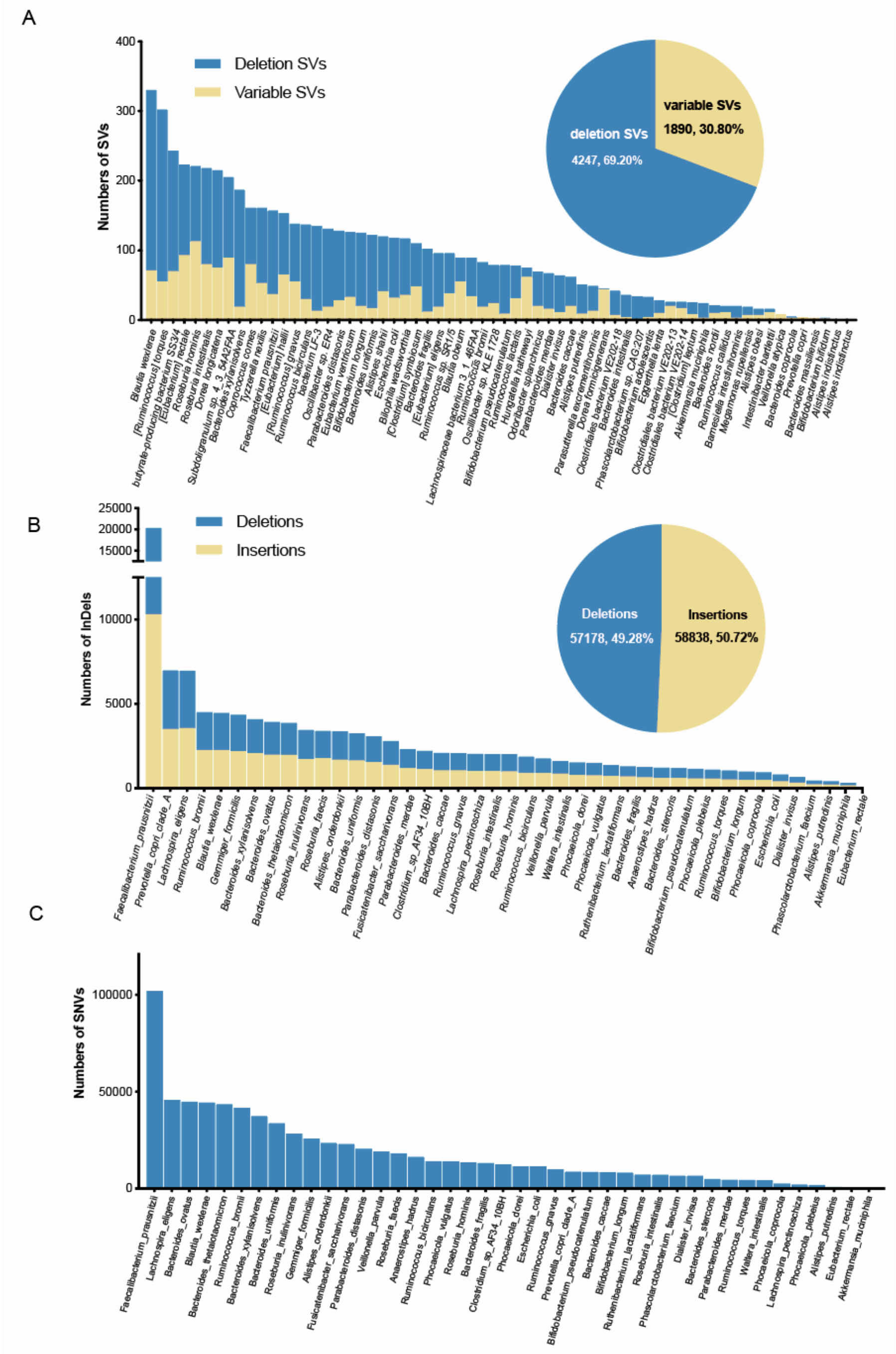

Supplementary figure 2 | Genomic variants profile of the gut microbiome. Related to Figure 2. (A) Number of detected structural variants (SVs), including deletion SVs (dSVs) and variable SVs (vSVs), across 63 species. The pie chart shows the proportion of dSVs and vSVs among all annotated SVs. (B) Number of detected InDels across 42 species. Pie chart indicates the proportion of insertions and deletions among all annotated InDels. (C) Number of SNVs identified across 42 species.

Chen et al. Supplementary figure 3

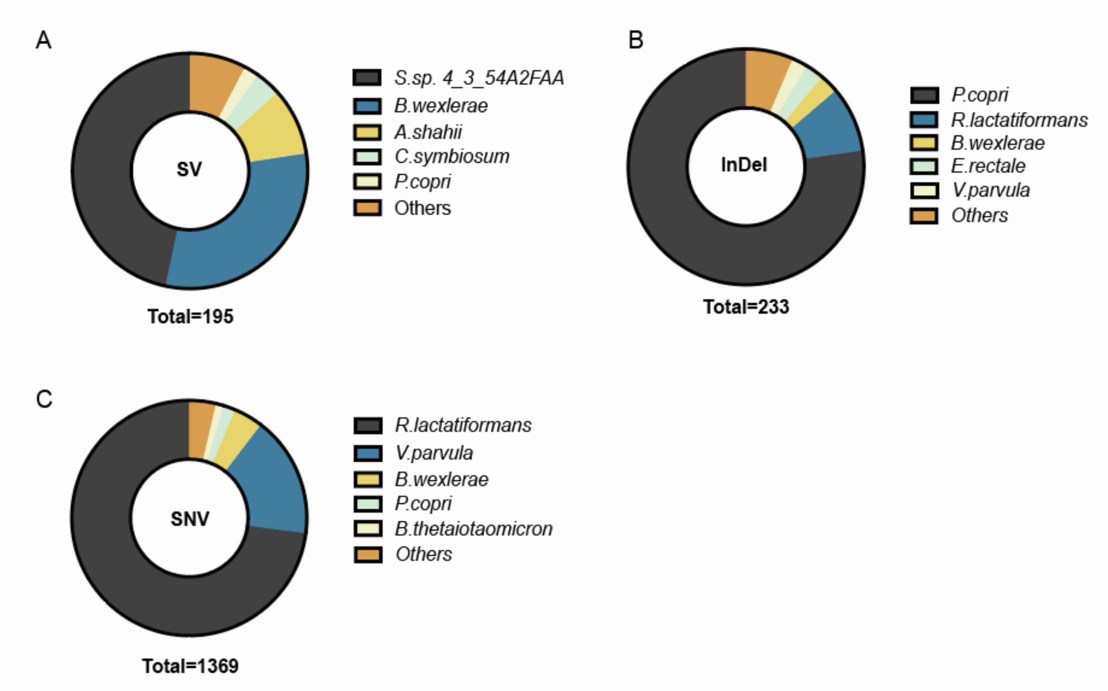

Supplementary figure 3 | Species distribution of differential genomic variants. Related to Figure 2. (A–C) Pie charts showing the distribution of species harboring differential SVs (A), InDels (B), and SNVs (C), respectively.

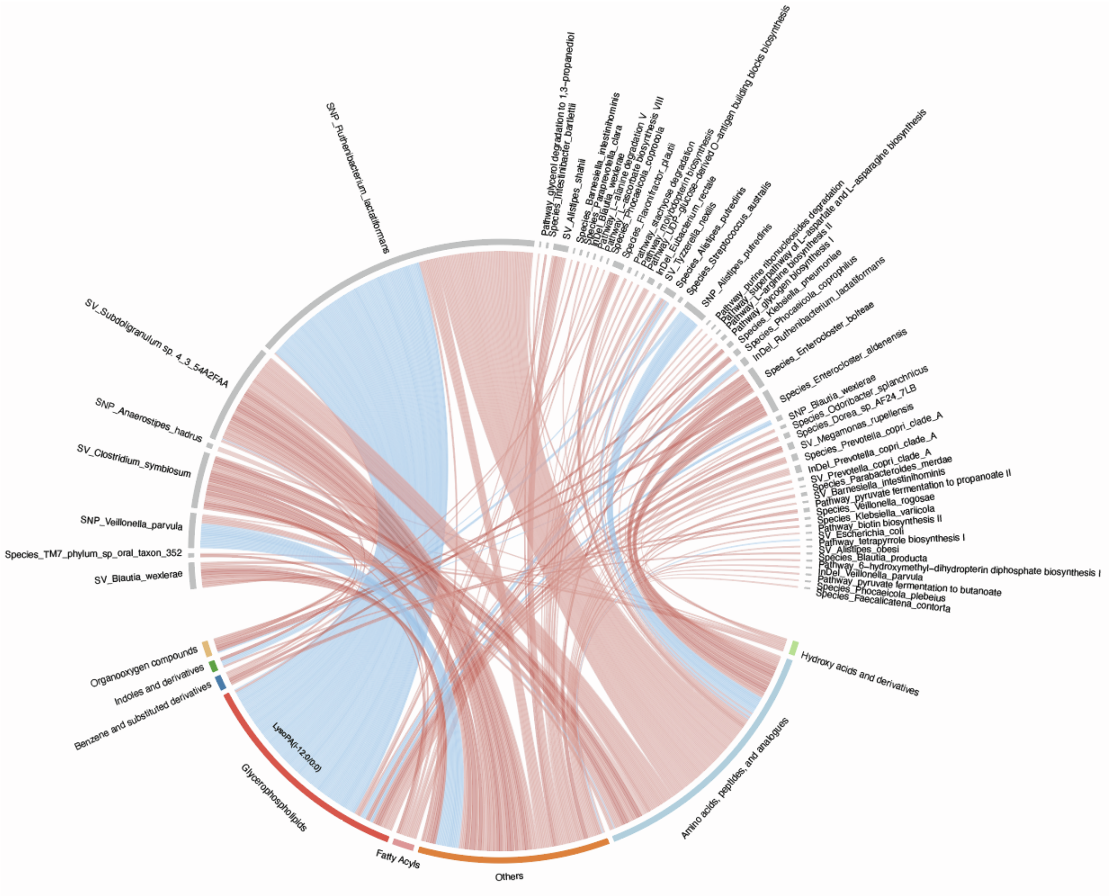

Supplementary figure 4 | Significant associations between fecal metabolites and microbial signatures. Related to Figure 5. Chord diagram illustrating significant correlations between microbial features and fecal metabolites. Associations in the upper semicircle are grouped by microbial species, while those in the lower half are grouped by metabolite categories. Red chords represent positive correlations; blue chords represent negative correlations.

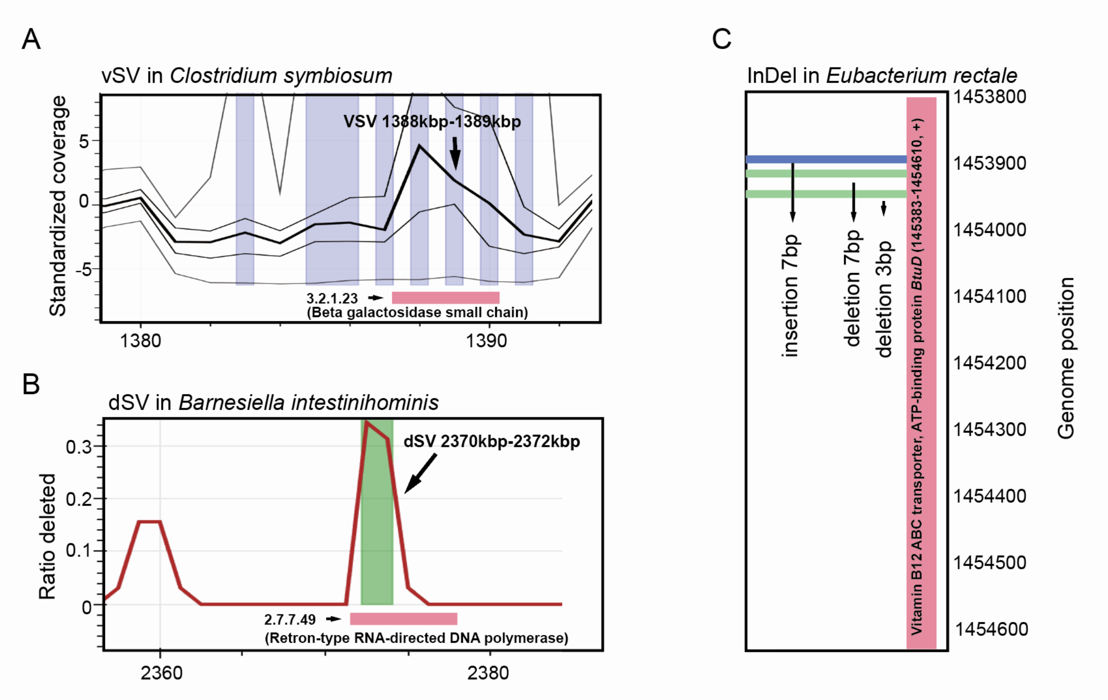

Supplementary figure 5 | Examples of differential SVs or InDels related to metabolites. Related to Figure 5. (A) Purple rectangles with arrows indicate vSVs significantly associated with multiple metabolites such as γ-Glutamylleucine and L-Glutamic acid. The vSV affects *beta-galactosidase*, a key enzyme involved in microbial carbohydrate metabolism. (B) Green rectangles with arrows denote differential dSVs positively associated with trans-3-Hydroxycotinine. These variants disrupt retron-type RNA-directed DNA polymerase, which may be involved in bacterial stress responses and phage defense mechanisms. (C) Schematic representation of three indels located in the genomic region encoding the vitamin B12 ABC transporter (*BtuD*), showing positive associations with fecal levels of caffeine and L-glutamine. Blue and green rectangles indicate insertions and deletions, respectively.

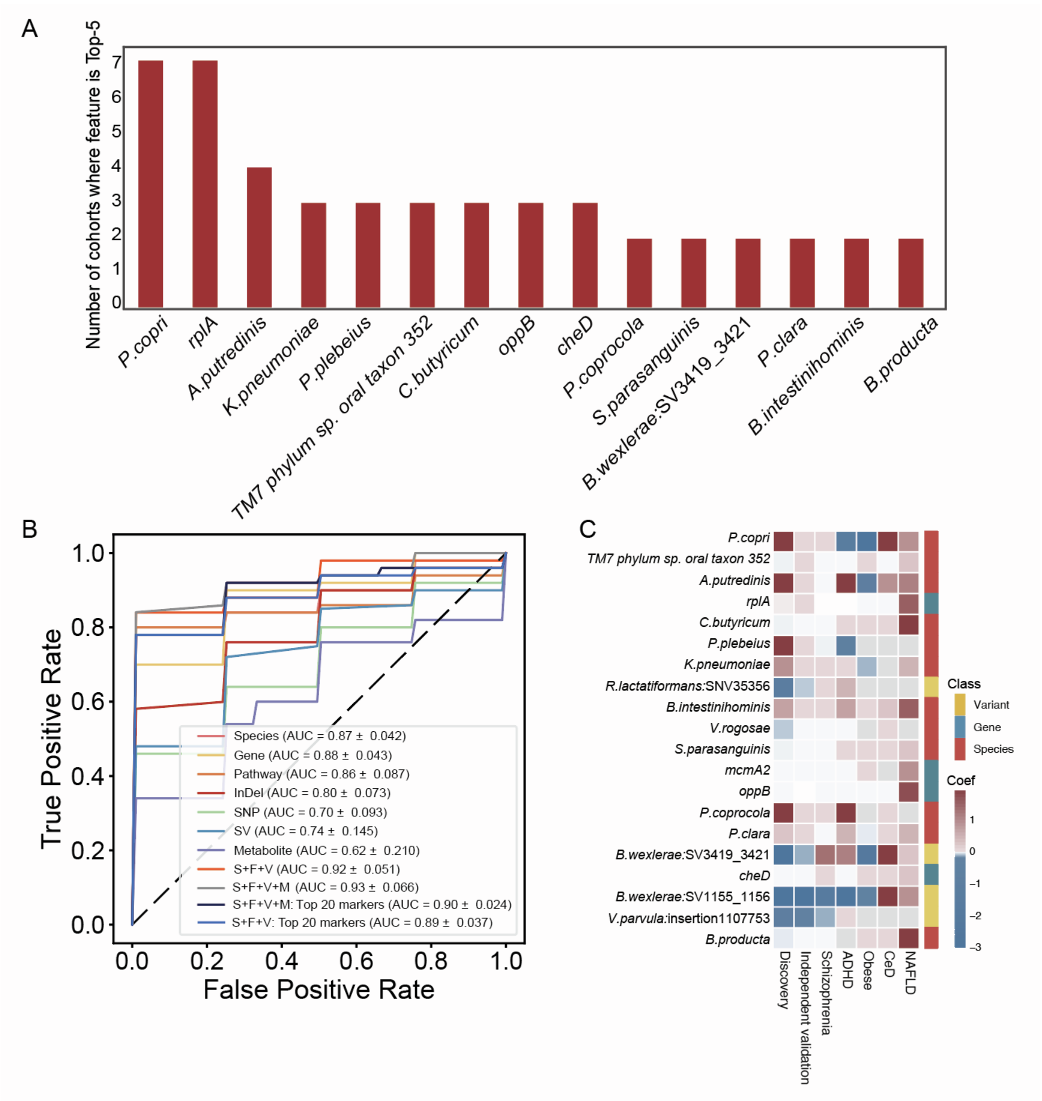

Supplementary Figure 6 | Validation of diagnostic models across feature types and cohorts. Related to Figure 6. (A) Frequency of each feature appearing among the top five contributors (by mean absolute SHAP value) across 10 cohorts (1 discovery + 9 external validation). (B) ROC curves showing the performance of diagnostic models based on different feature types in the independent in-house validation cohort. Models were constructed using species-level profiles (S), microbial gene and pathway functions (F), genomic variants (V; including SVs, InDels, and SNVs), and fecal metabolites (M). (C) Distribution of the top 20 minimal marker panel (based on metagenomic features) across external validation cohorts. Coef, coefficient from MaAsLin2.

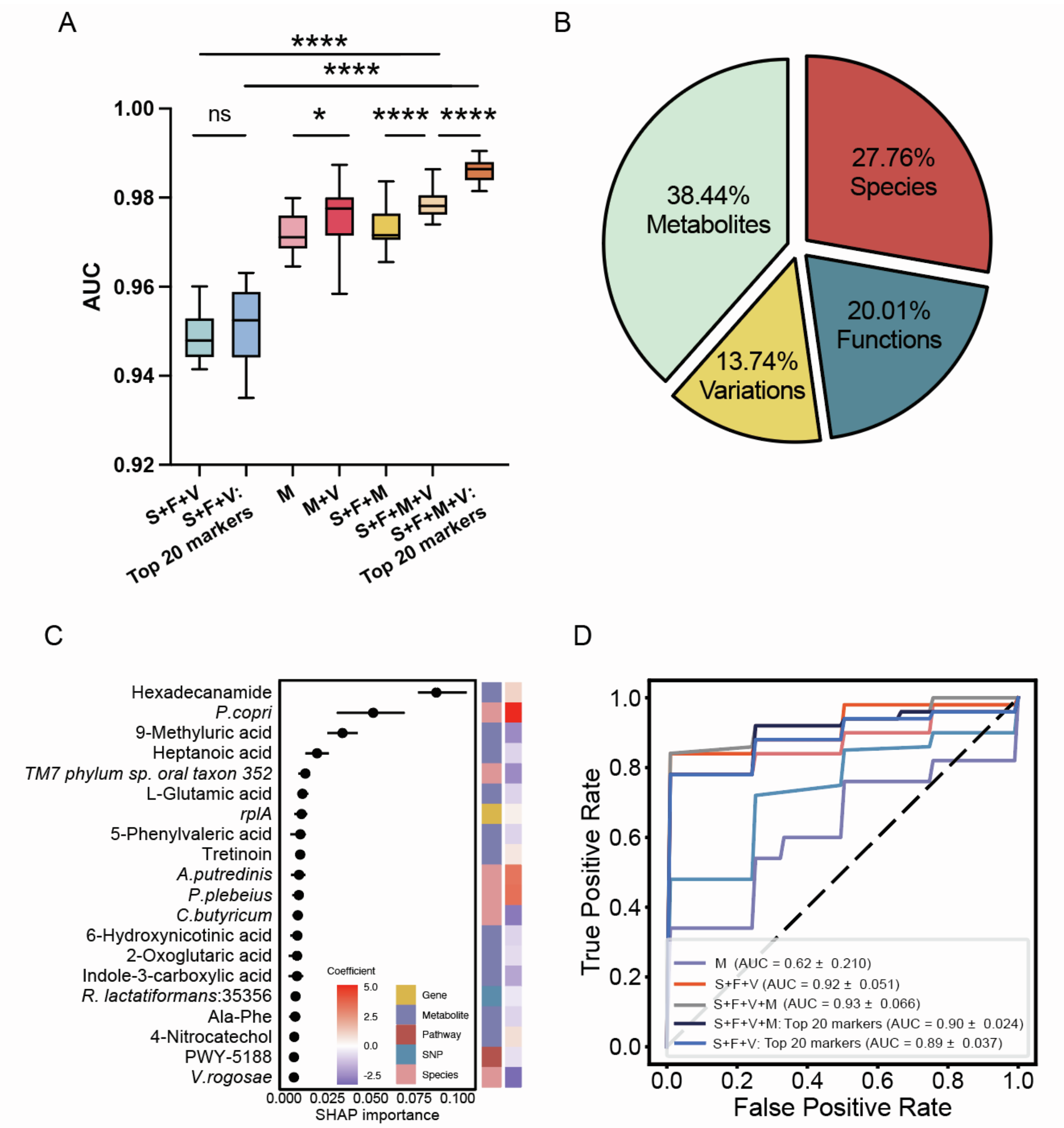

Supplementary Figure 7 | The diagnostic models of ASD based on multi-omics biomarkers. Related to Figure 6. (A) Comparison of model performance using metabolic features alone versus models additionally incorporating other data. P values were calculated using two-sided Wilcoxon rank-sum tests. *, $p < 0.05$; ****, $p < 0.0001$; S, species; F: microbial gene and pathway functions; V: genomic variants, including SVs, InDels, and SNVs; M: fecal metabolites. (B) Relative contribution of each feature type in the integrated model combining four data types, calculated using group-normalized SHAP (SHapley Additive exPlanations) values, where the summed contribution of each feature class was normalized by the number of features in that class. (C) Feature importance of the 20-marker minimal panel. Feature types and their direction of change in ASD are indicated. (D) Validation of the model in the in-house independent validation cohort.

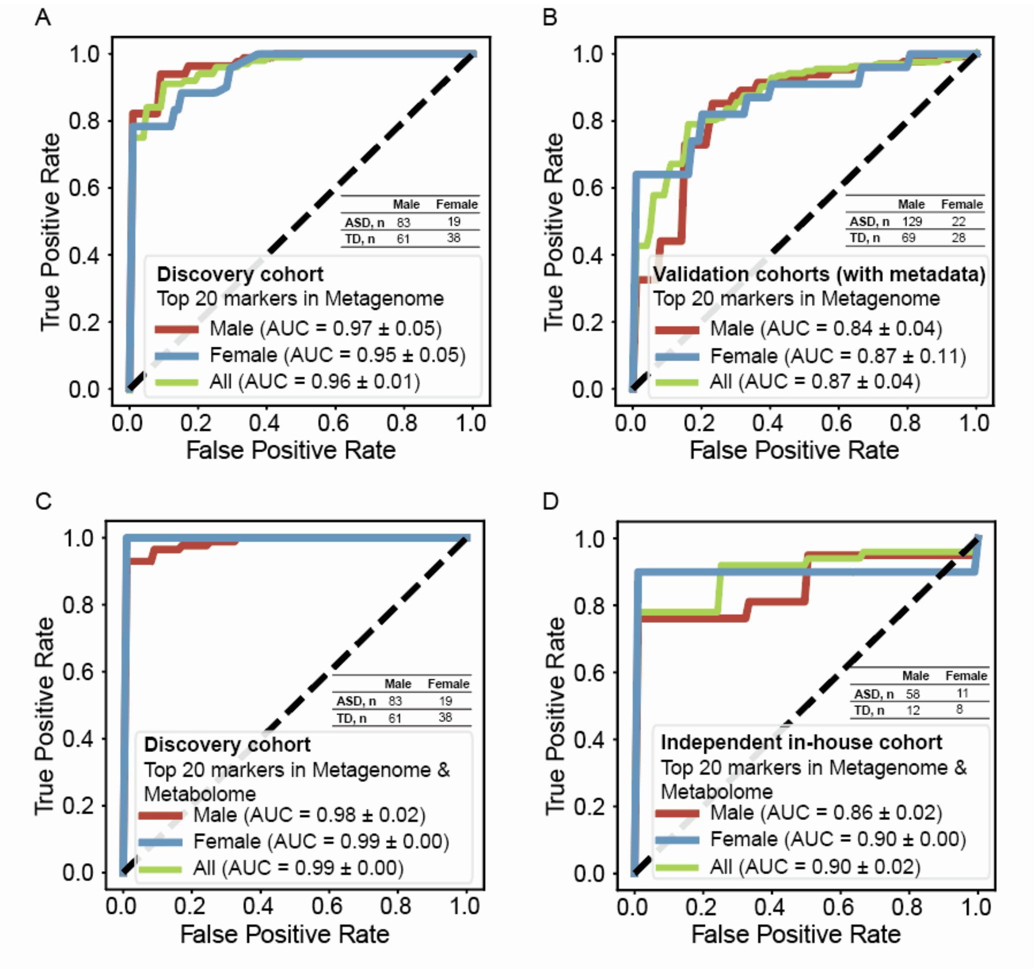

Supplementary figure 8 | Model performance stratified by gender. Related to Figure 6. Stratified analyses were conducted in the discovery cohort and in external validation cohorts with available gender information, including our independent validation cohort, PRJEB23052, PRJNA451479, and PRJNA516054 (248 samples in total). (A–B) Diagnostic performance of the top 20-marker microbiome-only model in males and females from the discovery cohort (A) and from the external validation cohorts (B), shown as ROC curves and AUC distributions. (C–D) Diagnostic performance of the top 20-marker integrated microbiome–metabolome model in males and females from the discovery cohort (C) and from the independent in-house validation cohort (D), shown as ROC curves and AUC distributions.
